# Mitochondria and Their Relationship with Common Genetic Abnormalities in Hematologic Malignancies

**DOI:** 10.3390/life11121351

**Published:** 2021-12-07

**Authors:** Ibolya Czegle, Austin L. Gray, Minjing Wang, Yan Liu, Jun Wang, Edina A. Wappler-Guzzetta

**Affiliations:** 1 Department of Internal Medicine and Haematology, Semmelweis University, H-1085 Budapest, Hungary; czibolyka@gmail.com; 2 Department of Pathology and Laboratory Medicine, Loma Linda University Health, Loma Linda, CA 92354, USA; agray@llu.edu (A.L.G.); yanliu@llu.edu (Y.L.); JWang@llu.edu (J.W.); 3 Independent Researcher, Diamond Bar, CA 91765, USA; minjing2007@yahoo.com

**Keywords:** hematological malignancies, genetic abnormalities, mitochondria, metabolism, fission-fusion, therapeutic targets

## Abstract

Hematologic malignancies are known to be associated with numerous cytogenetic and molecular genetic changes. In addition to morphology, immunophenotype, cytochemistry and clinical characteristics, these genetic alterations are typically required to diagnose myeloid, lymphoid, and plasma cell neoplasms. According to the current World Health Organization (WHO) Classification of Tumors of Hematopoietic and Lymphoid Tissues, numerous genetic changes are highlighted, often defining a distinct subtype of a disease, or providing prognostic information. This review highlights how these molecular changes can alter mitochondrial bioenergetics, cell death pathways, mitochondrial dynamics and potentially be related to mitochondrial genetic changes. A better understanding of these processes emphasizes potential novel therapies.

## 1. Introduction

The contribution of mitochondria in hematologic malignancies as possible targets for therapy-resistant malignancies is emerging and has been discussed in some excellent recent review articles, such as the one by Barbato and co-workers [1]. The attention is not surprising, given the need for new therapeutical targets in therapy-resistant cases and due to the diverse role of mitochondria in normal and tumor tissue. Mitochondria are not just essential in ATP production via oxidative phosphorylation (OXPHOS) (see all the Abbreviations in Table S1); it is also vital in other biosynthetic and bioenergetic pathways, apoptosis regulation, intracellular calcium and reactive oxygen species (ROS) signaling, and iron storage, metabolism, and heme biosynthesis [2,3,4,5]. Furthermore, in the last two decades, our understanding of mitochondrial fission, fusion, mitophagy, and mitochondrial trafficking has largely evolved, giving us more insight into their function in health and disease [2,3].

In this review article, we will focus on our current understanding of the role of mitochondria in health and disease, with a focus on their role in carcinogenesis. This will be followed by their role in hematologic malignancies, organized and discussed by the different diseases or disease groups. In each disease or disease group, we will highlight how their common genetic abnormalities are related to mitochondria and what additional therapeutic potential they hold. These genetic changes or chromosomal abnormalities are either driver mutations or have prognostic value, often related to poor prognosis or therapy resistance in a certain disease. The genes we discuss are chosen using the current literature and the latest (2017) WHO Classification of Hematopoietic and Lymphoid Tissue Malignancies book as a guide [6], latter which we use in practice for the diagnoses of these diseases.

## 2. Mitochondria in Healthy and Tumor Cells

Many mitochondrial changes are advantageous for cellular adaptation and proliferation, making them essential elements of cancer cell survival. These alterations, therefore, serve as potential therapeutic targets in various tumors. In this chapter, we discuss the main mitochondria-related changes concerning carcinogenesis.

### 2.1. Oxidative Phosphorylation (OXPHOS) and Reactive Oxygen Species Production

#### 2.1.1. Mitochondrial Metabolism 

Mitochondria have a complex role in cellular metabolism, producing adenosine triphosphate (ATP) during OXPHOS; or from using NADH and FADH2, generated via cytoplasmic β-oxidation of fatty acids or the tricarboxylic acid cycle (TCA), latter also known as Krebs cycle. In addition, mitochondria are responsible for synthetizing lipids, amino acids, pyrimidine, and various other metabolic intermediates necessary for a functioning cell [4].

#### 2.1.2. OXPHOS and Anaerobic Glycolysis

During OXPHOS, electrons are delivered through the mitochondrial respiratory complexes, making a proton gradient across the inner mitochondrial membrane, which is the source of ATP production during this process. OXPHOS requires the presence of oxygen and often generates ROS, especially at complexes I and II [3]. On the other hand, anerobic glycolysis takes place in the cytosol, not requiring oxygen. It produces lactate while generating less ATP than OXPHOS. Lactate can also be converted to pyruvate by lactate dehydrogenase (LDH), which can then enter the mitochondria to generate ATP through OXPHOS [7,8].

#### 2.1.3. Mitochondrial Metabolism Adaptation

Mitochondrial metabolism adapts to different environmental stresses, making the cell capable of surviving under ever-changing conditions. Tumor cells, in general, have unique ways to maintain their high energy demand, with mitochondria in the center of reprogramming their metabolism [7]. Interestingly, tumor cell ATP production shifts to primarily increased glycolysis with ongoing OXPHOS, which can be reduced, normal, or increased [7,9]. The original observation that cancer cells use significantly more glucose and produce a large amount of lactate was made by Otto Warburg in the 1920s [10]. This finding is since referred to as the “Warburg effect” [7,11,12]. The reason why these changes are advantageous to tumors is diverse. First, anaerobic glycolysis can be 10–100 times faster than OXPHOS. In addition, glycolysis can be advantageous when the tumor cells compete for energy sources and oxygen. Glycolysis also can support amino acid and nucleic acid synthesis, essential for cell proliferation [11]. In addition, tumor cells can increase the efficacy of their glucose transporter, such as GLUT1, to import more glucose to the cytoplasm [13]. However, OXPHOS is still important in tumor cell metabolism, with many tumor cells having normal or increased OXPHOS along with accelerated glycolysis [12]. Increased OXPHOS has been linked to more aggressive tumor behavior [8].

#### 2.1.4. Mitochondrial Metabolism as a Therapeutic Target

Based on these findings, mitochondrial metabolism has been proposed as potential tumor therapy in addition to inhibiting glycolysis. Several inhibitors are available in targeting mitochondrial metabolic pathways or enzymes, such as the ones in the electron transport chain (ETC) (metformin, ME344, IACS10759), enzymes in the TCA cycle (devimistat, enasidenib, ivosidenib), or glutaminase (converting glutamine to glutamate) (CB839, compound 27) [7,14]. As discussed later in this article, several of these pathways are affected by genetic alterations seen in hematologic malignancies. The ETC inhibitor ME344 has been shown to reduce acute myeloid leukemia (AML) cell viability and cell growth with no effect on normal hematopoietic cells [7,15]. In addition, inhibitors of a defective TCA cycle enzyme, the isocitrate dehydrogenase (AG120/ivosidenib, and AG221/enasidenib), have been FDA-approved for treating IDH-mutated relapsed/refractory AML [7,8,16].

#### 2.1.5. ROS Generation and Function

As previously mentioned, OXPHOS is important in the production of ROS. Low levels of ROS are constantly produced in normal cells when electrons escape from the ETC, typically from complexes I and II, and react with molecular oxygen. ROS are important in intracellular signaling in low levels, such as in the response to hypoxia, growth factor-induced cell proliferation, and inflammatory response generation [3,9]. In addition, a moderate amount of ROS can activate intracellular signaling pathways critical in tumor cell survival, such as mitogen-activated protein kinase/extracellular signal-regulated protein kinases 1/2 (MAPK/ERK1/2), p38, c-Jun N-terminal kinase (JNK), and phosphoinositide-3-kinase/protein kinase B (PI3K/Akt) [17]. Given that higher levels of ROS would result in severe damage and eventually in apoptotic or necrotic cell death, a well-developed anti-oxidant system is in place to remove these highly reactive molecules [18]. Other than cell death induction, increased ROS production often leads to DNA mutations, especially in the mitochondrial DNA (mtDNA), given its proximity to the ETC proteins, and the minimal amount of mtDNA repair mechanisms existing. These mutations eventually can lead to carcinogenesis and tumor progression [19].

#### 2.1.6. ROS in Tumor Therapy

**Figure 1:**
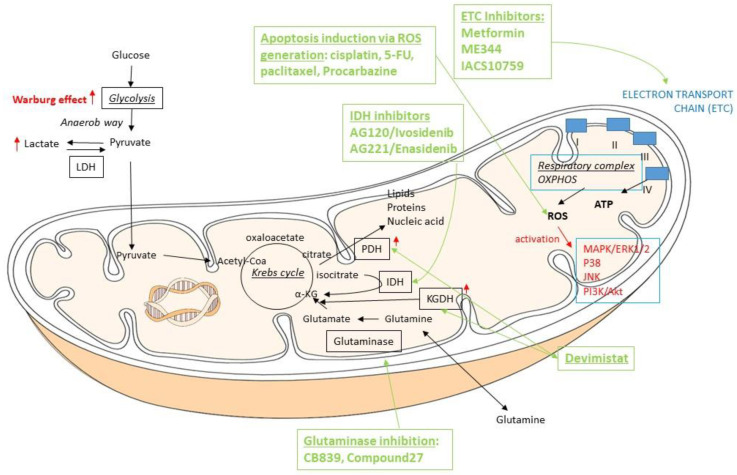
The adaptation of mitochondrial metabolism in cancer cells. Black arrows: biochemical processes under normal circumstances. Red text and arrows: the mechanism of dysregulation in tumor tissue. Green text and arrows: possible therapeutic targets. Abbreviations: ATP: adenosine trisphosphate, ROS: reactive oxygen species, ETC: Electron transport chain. PDH: pyruvate dehydrogenase, KGDH: α-ketoglutarate dehydrogenase, IDH: isocitrate dehydrogenase, α-KG: α -ketoglutarate.

Most tumor cells, including leukemia cells, produce an increased amount of ROS due to their increased metabolism and, therefore, can be targeted with ROS-generating agents to induce apoptosis [18]. Drugs using this mechanism of action are some widely used cytotoxic chemotherapies, such as cisplatin, 5-fluorouracil and paclitaxel. In addition, the ROS-producing anti-cancer drug procarbazine, has been approved for the treatment of stage III and IV Hodgkin lymphoma [7,14,20] and have been shown to be effective in other kinds of tumors as well [7]. The mitochondrial adaptation in carcinogenesis is summarized in Figure 1.

### 2.2. Mitochondrial DNA (mtDNA)

#### 2.2.1. mtDNA

The human mtDNA are multi-copied and have circular genome, approximately 16 kb in size, encoding 13 mRNA, 22 transfer RNAs (tRNAs), and two ribosomal RNAs (rRNAs). The coding regions in the mtDNA are in close connection with partial overlaps. One longer non-coding region is present in the mtDNA, referred to as the control region, with transcription promoters for both strands. The 13 encoded proteins are essential subunits of the ETC I, II, IV, and V, with the majority of the mitochondrial proteins encoded by the nuclear DNA (nDNA). The nuclear-encoded mitochondrial proteins are imported from the cytosol by two translocase complexes: the translocase of the outer membrane (TOM) and the translocase of the inner membrane (TIM) [4,21,22,23,24].

#### 2.2.2. mtDNA Transcription 

Mitochondrial biogenesis requires coordinated nuclear and mitochondrial gene expressions for effective protein synthesis, regulated by various transcription factors. Several different proteins can directly or indirectly regulate mtDNA transcription. Most of those directly binding to the mtDNA bind to the D-loop area in the control region. The direct regulators include the mitochondrial RNA polymerase (POLRMT), transcription factors mitochondrial transcription factor A (TFAM), and mitochondrial transcription factor B1/2 (TFB1M/2M), a transcription elongation factor (TEFM), and one termination factor (mTERF1). TFAM can stabilize the mtDNA and initiate transcription, and is regulated by many intracellular regulatory proteins, such as the protein kinase A (PKA), extracellular regulated protein kinases 1/2 (ERK), or the peroxisome proliferator-activated receptor gamma coactivator 1-α (PGC-1α). A small change in PGC-1α’s concentration strongly affects mtDNA replication and transcription, and it is involved in mtDNA copy number alteration, mitochondrial dynamics, and OXPHOS regulation. Other molecules, such as signal transducers and activators of transcription 3 (STAT3) and cAMP response element-binding protein (CREB) can also bind to the D-loop area, promoting TFAM-independent gene expression. Other transcription enhancers, such as c-Jun and CCAAT enhancer-binding protein (CEBP)β, bind to different mtDNA regions [21,24,25,26]. In addition, *POLG* and *POLG2* genes, encoding mtDNA polymerse γ, are involved in mtDNA replication, whereas *TWNK* encodes twinkle mtDNA helicase [27].

#### 2.2.3. mtDNA Damage and Repair 

mtDNA is more vulnerable to insults, such as ROS production, than nDNA due to its proximity to the ROS production site, smaller amount of repair mechanisms existing, and the lack of protection by histones [23]. Replication errors, however, also contribute to a large number of mtDNA mutations [25]. mtDNA repair mechanisms are not well characterized, and the damaged mtDNA can also be degraded. Repair mechanisms include base excision repair, and direct reversal, with evidence of mismatch repair and double-strand break repair possibly existing in the human mitochondria [23,28]. Importantly, mtDNA mutations can be heteroplasmic or homoplasmic, indicating the presence of a mixture or identical mtDNA genotypes in the mitochondria, respectively [7].

#### 2.2.4. mtDNA Translation as a Therapeutic Target

Inhibitors of mitochondrial transcription at the *POLRMT* level have been developed, such as IMT1 and IMT1B. They successfully decrease OXPHOS and ATP production via interfering with the transcription of ETC proteins. In addition, these inhibitors reduced tumor cell growth and cell viability in ovarian and colon cancer models [29]. Mitochondrial protein translation can also be inhibited by drugs, such as Tigecycline, which could promote cell death in AML cells, and can improve the effectiveness of tyrosine kinase inhibitor Imatinib in chronic myeloid leukemia (CML) models [7,30,31].

#### 2.2.5. mtDNA Changes in Cancer

**Figure 2:**
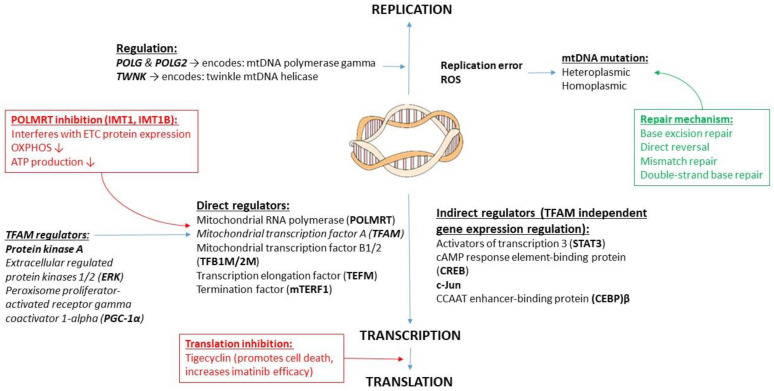
The regulation of mtDNA replication, transcription and translation and their role as therapeutic targets. Blue arrows: mtDNA replication, transcription, translation, and their regulators. Red text and arrows: inhibitors. Green text and arrow: mtDNA repair mechanisms.

Not only are mtDNA mutations more frequent in cancer cells, but altered mtDNA copy number has also been associated with carcinogenesis [7,32,33,34]. While some types of cancer cells exhibit decreased mtDNA copy number, lymphoma and certain leukemia cells are typically associated with increased mtDNA copy number [32,35,36]. In pediatric AML cells increased *TFAM* and *POLG* expression have been described along with increased mtDNA copy number, reversible with PGC-1α inhibition [35]. In addition, mtDNA copy number was found to be increased in pediatric acute lymphoblastic leukemia (ALL) samples, which significantly decreased after treatment [37]. Additionally, there is growing evidence that mtDNA polymorphisms can influence drug response in various cancers [38]. The regulation of mtDNA replication, transcription and translation are summarized in Figure 2.

### 2.3. Apoptosis and Necrosis Regulation

Apoptotic pathways and their role in carcinogenesis have been well studied. It can be initiated via the extrinsic or intrinsic pathway leading to caspase activation, the central effectors of apoptotic cell death [22]. The intrinsic pathway is activated by intracellular stress signals, which trigger the homo-oligomerization of bak and bax proteins, creating pores on the mitochondrial outer membrane (MOM) and releasing cytochrome c from the mitochondrial intermembrane space. Bak and bax, along with PUMA and NOXA are pro-apoptotic members of the B-cell lymphoma 2 (Bcl 2) protein family, which are inhibited under normal circumstances. Anti-apoptotic members of the Bcl-2 protein family, such as bcl-2, bcl-Xl, and mcl-1, can bind and inhibit bak and bax in their inactivated or activated forms. Cytochrome c induces a so-called apoptosome formation, able to activate caspase 9, which in turn can activate the executioner caspase, caspase 3 [39,40,41]. Interestingly, the most famous anti-apoptotic protein in cancers, p53 has a much more complex effect on mitochondria than just interfering with the apoptotic pathways, described in more details in Section 3.5.2. The extrinsic pathway of apoptosis is initiated via the activation of a death receptor, which activates caspase 8 and then caspase 3 [22,39].

#### 2.3.1. Necrosis and Necroptosis

Unlike apoptosis, necrosis is an unregulated process of cell death caused by severe injury. However, necroptosis, a controlled form of necrosis was described in 2005 by Degterev and co-workers [42], which has been further characterized since [39]. The main control proteins of necroptosis are the receptor-interacting proteins 1 (RIP1) and 3 (RIP3), activated in an apoptosis-deficient environment. Interestingly, RIP1 can induce apoptosis via caspase 8 activation, but it also initiates necroptosis via recruiting RIP3. RIP3 can interact with the mixed lineage kinase domain-like pseudokinase (MLKL) protein, which becomes an oligomer after the interaction and migrates to the cell membrane, increasing its permeability [39].

#### 2.3.2. Apoptosis as a Therapeutic Target

The connection between the pro- and anti-apoptotic genes and carcinogenesis is well described in the literature. Increased expression of anti-apoptotic proteins, such as bcl-2 and bcl-XL are seen in untreated AML samples and advanced MDS, with higher expression associated with worse prognosis or therapy resistance. A Bcl-2 inhibitor, venetoclax (ABT-199), has been used to treat AML and relapsed Chronic lymphocytic leukemia (CLL) with 17p deletion. Similarly, altered anti-apoptotic protein mcl-1 expression was described in hematologic malignancies, such as AML, multiple myeloma (MM), and B-cell acute lymphoblastic leukemia (B-ALL). Conversely, bax level can be high with either therapy sensitivity or therapy resistance in AML. Their localization is, however, important. The cells are more sensitive to chemotherapy when bax is mainly in the mitochondria, and not in the cytosol. Interestingly, BTSA1, a bax activator, effectively suppress apoptosis in human AML xenografts [41,43,44,45].

#### 2.3.3. Necroptosis as a Therapeutic Target

Necroptosis is also a potentially viable treatment for cancers that have acquired resistance to apoptotic pathways. The serine-threonine kinases RIP1 and RIP3 have been shown to induce mitochondrial fission through activation of dynamin-related protein1 (Drp1) via serine phosphorylation. Subsequently, Drp1 can interact with another regulator protein, Fis1, to induce mitochondrial fission. Ultimately, RIP1-RIP3 induced mitochondrial fission will lead to the generation of reactive oxygen species and eventually cell death via necroptosis [46]. Necroptosis, however, can also occur independently of mitochondrial fission, which might be cell-type dependent [47].

### 2.4. Mitochondrial Fission and Fusion, Mitophagy

#### 2.4.1. Mitochondrial Dynamics

The changes in mitochondrial number and morphology are described as mitochondrial dynamics [41]. Mitochondria are highly dynamic structures that form a cytosol network, responsive to intracellular changes, such as altered metabolic needs. The adaptation of the mitochondrial network is via changes in mitochondrial dynamics, including fission, fusion, and “mitophagy”. These processes regulate the number of mitochondria in the cells, and they are also important in the redistribution or elimination of mtDNA. In addition, autophagic degradation of the mitochondria, known as “mitophagy”, is responsible for removing healthy mitochondria when less is needed or damaged mitochondria to maintain a healthy pool in the cell [2,23,48,49].

#### 2.4.2. Mitochondrial Fission

Mitochondrial fission results in an increased number of mitochondria, with dynamin-related protein 1 (Drp1), a large GTPase, being the central effector protein [50,51]. Drp1 is primarily found in the cytosol and is translocated to the MOM at the time of fission [49,51] to any of its mitochondrial receptors: fission protein 1 (Fis1), mitochondrial fission factor (Mff), or mitochondrial elongation factors (MIEFs) 1 and 2. Amongst the Drp1 receptors, Fis1 is the least significant in mammals [49]. When Drp1 is recruited to the mitochondrial surface, it polymerizes and forms a ring-like structure around the outer membrane of the mitochondria, where fission occurs via its GTPase activity [49]. Mitochondrial fission results in changes in mitochondrial metabolism, most notably in decreased OXPHOS capacity [52]. An increase in Drp1 and fission occurs due to different stress mechanisms, and as a part of cell adaptation mechanisms. A significant decrease or loss of mitochondrial outer membrane potential (MOMP) of damaged mitochondria for example results in mitochondrial fragmentation. Interestingly this can be due to increased fission but can also result in shortened mitochondria due to the collapse of the mitochondrial structure [5]. Fission is also an important part of removing aberrant mitochondria via selective mitophagy, such as following uneven fission, when one daughter mitochondrion with low MOMP would be eliminated [25].

#### 2.4.3. Mitochondrial Fusion 

On the other hand, mitochondrial fusion results in a decreased number, and more interconnected mitochondria, with increased oxidative capacity and MOMP [25,52]. Key proteins involved in mitochondrial fusion are mitofusin 1 (Mfn1) and 2(Mfn2) on the MOM and optic atrophy 1 (OPA1) on the inner mitochondrial membrane (IMM). Both Mfn1 and 2 are transmembrane proteins with GTPase activities that can form homotypic (Mfn1-Mfn1 or Mfn2-Mfn2) or heterotypic (Mfn1-Mfn2) connections to fuse the outer mitochondrial membranes of two mitochondria, while the homotypic Opa1-Opa1 complexes fuse the internal mitochondrial membranes [25,48,53]. Additionally, Mfn2 is involved in the endoplasmic reticulum (ER)—mitochondria tethering; in mitophagy, via ubiquitination (see later in this chapter); and in apoptosis regulation (see later in this chapter) [22]. Damaged mitochondria with reduced MOMP are unlikely to undergo fusion, partially due to decreased OPA1 levels, which helps to eliminate and isolate those mitochondria from the healthy pool. In addition, higher OPA1 expression is linked to decreased mitophagy [25,52].

#### 2.4.4. Mitophagy

Besides clearing dysfunctional mitochondria, mitophagy is important in various adaptive responses, where reduced overall mitochondrial mass is desirable [25], and is also involved in controlling inflammatory responses in immune cells [54]. The major pathways of selective mitophagy are the ubiquitin-mediated and the receptor-mediated mitophagies, with evidence of significant crosstalk between them [54,55,56]. Ubiquitin-mediated mitophagy can be parkin-dependent or parkin-independent. The parkin-dependent PINK1-parkin pathway is the classic and most studied pathway of mitophagy. It eliminates defective organelles, which have for example reduced MOMP. Reduced MOMP results in an increased number of the phosphatase and tensin homolog (PTEN)-induced putative kinase 1 (PINK1) protein on the outer mitochondrial membrane (OMM), as the unfunctional mitochondria cannot transport PINK1 to the IMM to be cleaved [55,56]. Therefore, in mitochondrial membrane depolarization, mitochondrial complex dysfunction, mtDNA mutations, or proteotoxicity, PINK1 accumulates at the OMM rapidly and recruits parkin from the cytosol via phosphorylation. Activated parkin ubiquitinates OMM proteins, such as VDAC1 and Mfn2, serving as an “eat me” signal for damaged organelles. The autophagy adaptor proteins, such as p62, Optn, and Ndp52 recognize these signals and initiate autophagosome formation. PINK1 can amplify the autophagy signals via phosphorylation of ubiquitin and poly-ubiquitin chains. PINK1 also increases Drp1 activity, resulting in the fragmentation of the damaged mitochondria, which can be cleared easier [25,53,55,56]. In addition to parkin, other ubiquitin E3 ligases can also trigger mitophagy via a PINK1-independent manner. These proteins include Gp78, SMURF1, SIAH1, MUL1, and Arih1, which recruit autophagy adaptor proteins to complete the mitophagy process [56]. The receptor-mediated mitophagy pathway on the other hand is initiated by one of the mitophagy receptors, such as the Nip3-like protein X or Bnip3L (Nix), Bcl-2 family adenovirus E1B 19 kDa-interacting protein 3 (Bnip3), activating molecule in BECLIN1-regulated autophagy (ambra1) or FUN14 domain containing 1 (fundc1). These proteins can directly bind to LC3 in the phagophore, unlike in the non-receptor mediated pathway, where autophagy receptors are also needed for LC3 binding [25,54,55,57].

#### 2.4.5. Mitochondrial Dynamics in Different Cell Cycles

Mitochondrial fusion predominates G1/and early S-phases during the cell cycle, producing more ATP for cell growth. On the other hand, during S/G2/and M phases, mitochondrial fission is increased to provide equal distribution of mitochondria between daughter cells and to reduce OXPHOS and subsequent ROS generation and mtDNA mutations [52,58,59]. Blocking Drp-1 in vitro resulted in fewer cancer cells in S-phase and increased apoptosis [60,61].

#### 2.4.6. Mitochondrial Dynamics and OXPHOS

Both bioenergetic changes and apoptosis regulation via altered mitochondrial dynamics are important in carcinogenesis and thus hold tremendous therapeutic potential for various malignancies. As for the bioenergetic changes, fission generally reduces, whereas fusion increases OXPHOS efficacy [9,52]. Although both disrupted fission and fusion have been linked to impaired mitochondrial energy production in some cases, unopposed fusion has been shown to produce more ATP through enhanced cristae density and ATP synthase dimerization [62,63,64]. Therefore, the observation that mitochondrial dynamics are shifted towards fission in most tumors aligns with the decreased OXPHOS previously described in various cancer models [65].

#### 2.4.7. Mitochondrial Dynamics and Apoptosis

The relationship between apoptosis and mitochondrial fission/fusion is complex and bi-directional. First, both Drp1 and Mfn2 are related to the pro-apoptotic protein Bax. Both proteins can be co-localized with Bax at the MOM, with Drp1 being recruited by Bax after apoptosis induction, promoting fission; and Mfn2 binding to Bax, leading to mitochondrial fusion inhibition [52,53,66,67]. Furthermore, Drp1 plays an essential role in cytochrome c release and in pro-apoptotic Bak activation [51,68]. Downregulation or blocking of Drp1 has been shown to have anti-apoptotic effect in different in vitro models [53,68,69,70]. Of note, apoptosis can occur without mitochondrial fragmentation, with some studies showing that blocking fission only slows down apoptosis rather than entirely preventing it [53,68,71,72]. Fusion protein OPA1 on the other hand can inhibit cytochrome c release and resultant apoptosis [73,74]. Additionally, bak activation can induce mitochondrial fragmentation, whereas bcl-2 and bcl-Xl shift mitochondria to fusion [75]. Nevertheless, mitochondrial fission can induce apoptosis via increased ROS production [76].

Despite many models proving the pro-apoptotic effect of Drp-1 and mitochondrial fission, in most tumors, Drp1-mediated fission is anti-apoptotic. Drp1 expression is high in various tumors, which is associated with increased cell proliferation, metastatic potential, and poor outcome in many tumors, including AML [47,50,61,65,77,78,79,80]. Loss or inhibition of Drp1 was shown to inhibit tumor growth in multiple models [47]. Interestingly, the pro-apoptotic effect of the Drp1 receptor Fis1 was described, which seems to be unrelated to its fission function [58]. Blocking Mff has also been shown to inhibit tumor growth and cell proliferation in an in vivo model [81]. In addition, Mfn2 expression is decreased in several tumors, whereas increased Mfn2 expression has been linked to tumor suppression. In line with these findings, low Mfn2 expression in breast cancer patients is associated with poor outcome, and overexpression of Mfn2 was shown to induce apoptosis in a hepatocellular carcinoma model [65,82,83,84]. The explanation for the discrepancy in tumor cells versus other models is likely due to that increased mitochondrial fission in tumor cells can protect them from Ca^2+-^dependent apoptosis by limiting mitochondrial Ca^2+^ overload [58,85]. In addition, increased MOM surface impairs bax insertion and resultant bax-induced apoptosis [65].

#### 2.4.8. Mitophagy and Apoptosis

Mitophagy is also associated with apoptosis in varied ways. Excessive mitophagy induces apoptotic cell death [55]. The PINK1-parkin pathway can protect the cell from apoptosis via VDAC1 monoubiquitination, whereas it induces mitophagy via polyubiquitination of the same protein [86]. Mitophagy receptors Nix and Bnip3 have a BH3 domain similar to the Bcl-2 family proteins and can induce apoptosis [55,87].

#### 2.4.9. Mitochondrial Dynamics in Cancer

In sum, mitochondrial dynamics is a key regulator of apoptosis and cellular metabolism in cancer cells. Therefore, blocking Drp1-dependent mitochondrial fission, MFF, or increasing Mfn2 levels, increasing mitophagy receptor density on the mitochondria, or decreasing VDAC1 monoubiquitination are viable therapeutic options for several types of malignancies, including hematologic malignancies, as discussed later.

#### 2.4.10. Mitophagy in Cancer

**Figure 3:**
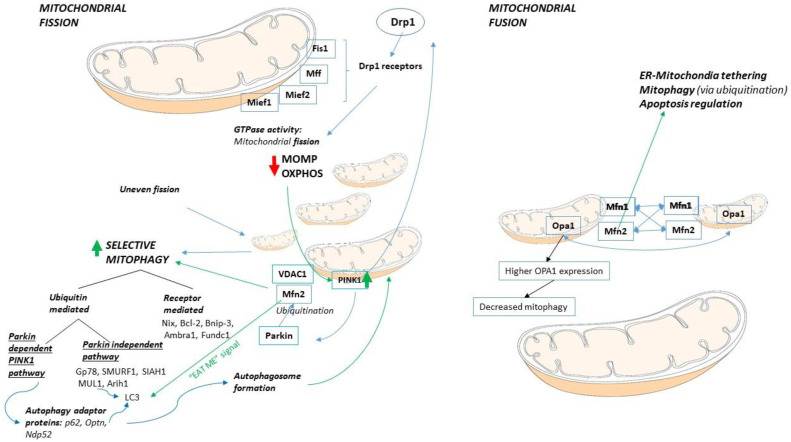
Mitochondrial fission-fusion, mitophagy and their regulation. Red arrows: inhibition. Green arrows: activation. Abbreviations: Drp1: Dynamin-related protein 1, Fis1: fission protein 1, Mff: mitochondrial fission factor, Mief: mitochondrial elongation factors, MOMP: Mitochondrial outer membrane potential, OXPHOS: oxidative phosphorylation, Mfn1,2: mitofusin 1,2, Opa1: Optic atrophy 1, PINK1: phosphatase and tensin homologue (PTEN)-induced putative kinase 1, Nix: Nip3-like protein X, Bnip3: adenovirus E1B 19 kDa-interacting protein 3, Ambra-1: activating molecule in BECLIN1-regulated autophagy, Fundc1: FUN14 Domain Containing 1.

Mitophagy is important both in normal hematopoietic cell development and in various hematologic malignancies, discussed in detail in a recent review by Stergiou and Kapsogeorgou [88]. In AML, decreased mitophagy was shown to reduce cell proliferation. Erythroblasts in low-risk MDS demonstrate increased mitophagy, whereas in the high-risk group, accumulation of enlarged mitochondria was present. In addition, mitophagy was found to be prominent in the myeloid lineage in MDS. Interestingly, mitophagy receptor, *BNIP3* and *NIX*, expressions were decreased in MDS, latter in the high-risk group [88,89,90,91,92,93]. Mitophagy inhibitors and inducers are listed in Section 4, Table 3. Mitochondrial dynamics are summarized in Figure 3.

### 2.5. Mitochondrial Trafficking

#### 2.5.1. Mechanisms of Mitochondrial Trafficking

Mitochondria travel in the cytosol via a microtubular network of β-tubulin or actin filaments. Mitochondria’s microtubular transport primarily uses Rho GTPases, such as Mitochondrial receptor protein 1 and 2 (Miro1/2), encoded by *RHOT1* and *RHOT2*, respectively. Miro1/2 binds to the kinesin-1/3 motor via the trafficking kinesin protein 2 (Trak2), encoded by the TRAK2 gene, moving the mitochondria towards the + end of the microtubule, called anterograde transport. The KIF5B gene encodes the kinesin-1 heavy chain. Besides Miro, additional kinesin-binding proteins, such as syntabulin (encoded by *SYBU*), fasciculation and elongation protein zeta 1 (encoded by *FEZ1*) and Ran-binding protein 2 (encoded by *RANBP2*), have been described, mostly in neurons and in some tumors of non-nervous system origin. For retrograde transport, Miro is forming a complex with a dynein motor and the bicaudal 2 (BiCD2) adaptor protein. There are four ways described in the literature on how mitochondrial detachment can occur from the microtubule when there is a mitochondrium-Miro-Trak2-kinesin complex present: 1. Myosin motor (MYO)-dependent binding to actin filaments 2. Mitochondrial anchoring to the microtubules via syntaphilin (SNPH), disrupting kinesin-Trak2 binding 3. Calcium-induced detachment resulting in conformational change in Miro and in the kinesin protein, and 4. The irreversible proteasomal degradation of the kinesin-1/Trak complex. All these can inhibit mitochondrial transfer along the microtubules. In addition, mitochondrial transfer along the actin filaments is typically short-range and is executed by MYOs, such as MYO19, MYO6, and MYO5. MYOs can move mitochondria both anterograde and retrograde, with the exact mechanism of mitochondrial binding unknown [50,52,94,95].

#### 2.5.2. Mitochondrial Trafficking in Cancer

Excellent recent review articles are available on the changes in mitochondrial trafficking in cancer [94,95,96], which we discuss here briefly. In recent years, changes in mitochondrial trafficking and mitochondrial localization have been described concerning cancer progression. Interestingly, the intracellular ATP:ADP ratio changes not only with a change in the mitochondrial mass but also with their position inside the cells [97]. Increased mitochondrial density, often with evidence of intense glycolysis at the plasma membrane has been described in invasive cells [96]. These changes likely help to have the energy source available right at the plasma membrane, supporting signal transduction and cell invasion [94]. Increased expression or mutation of genes involved in microtubule-based movements, such as *TRAK1*, *MIRO1/2*, *KIF5B*, *RANBP2*, or *FEZ1,* are associated with enhanced cell proliferation and invasion and chemotherapy resistance in some solid tumors. *SNPH* is typically downregulated in cancers, with its depletion or loss resulting in a worse prognosis and enhanced cell invasion. On the other hand, we have minimal knowledge on how the actin-MYO mitochondrial trafficking is altered in cancer cells. Furthermore, more research is needed to clarify how mitochondrial trafficking is affected in hematologic malignancies [94]. As discussed in later chapters, scattered data exist on their role in multiple myeloma (MM) and in MYC-related malignancies.

## 3. Mitochondrial Changes in Relation to Driver Mutations, Genetic and Chromosome Abnormalities in Hematologic Malignancies

### 3.1. Mitochondria in Hematologic Malignancies 

Primary mitochondrial issues in hematologic diseases, such as in Pearson syndrome or some congenital neutropenias, are rare [98,99]. In addition, according to a small study, primary mitochondrial disorders are not typically associated with hematologic malignancies but with anemia, thrombocytopenia, thrombocytosis, leukopenia, or eosinophilia [100]. Despite the fact that mitochondrial changes are not the root cause of hematologic malignancies, tumor cells heavily rely on them for their survival and therefore are great potential targets in their treatments [101]. Here we discuss these changes and treatment options by diseases and related genetic changes.

### 3.2. Myelodysplastic Syndrome (MDS) and Acute Myeloid Leukemia (AML)

#### 3.2.1. Myelodysplastic Syndrome (MDS)

MDSs are clonal hematopoietic malignancies predominating in people above 70 years. It is characterized by ineffective hematopoiesis leading to cytopenias and can transform to AML in approximately 30–40% of the cases. The etiology of MDS is unknown in 85% of the cases. Numerous cytogenetic alterations, hereditary or acquired mutations in somatic and mitochondrial genes, hereditary genetic syndromes, or environmental factors are responsible for developing the disease [102].

#### 3.2.2. Acute Myeloid Leukemia (AML)

AML is a phenotypically and genetically heterogeneous group of hematological malignancies characterized by clonal expansion of myeloid precursors with diminished capacity for differentiation [103]. AML represents 15–20% of acute leukemia cases in children and 80% in adults [104]. Population aging contributes to a significant increase in AML in Europe, as its incidence rises markedly in patients above 60 years. With advanced age, there is a decreased incidence of AML with recurrent genetic abnormalities [105]. In contrast, the incidence of other AML categories, such as AML with myelodysplasia-related changes (MRC-AML), or therapy-related AML (tAML), increases with age, comprising about 19% and 7% of AML cases, respectively [106,107,108,109]. According to the current World Health Organization (WHO) Classification of Tumors of Hematopoietic and Lymphoid Tissues, numerous genetic changes are highlighted, defining a distinct subtype of diseases (AML with recurrent genetic abnormalities), such as the ones involving *RUNX1*, *CBFB-MYH11*, *PML-RARA*, *BCR-ABL1* etc. Also, there is a separate category for myeloid neoplasms with germline mutations, including *DDX41* and *CEBPA,* amongst others. In addition, several genes are highlighted that provide prognostic information both in AML and MDS [6]. Additionally, various publications report further genetic abnormalities in correlation with prognosis.

#### 3.2.3. Cytogenetic and Molecular Genetic Alterations in MDS and MDS/AML

The most common genetic alterations in MDS/AML are clonal chromosomal abnormalities, found in approximately 30–80% of patients. In the remaining 20–70% of patients with normal karyotype, point mutations, microdeletions, amplifications, epigenetic changes, or copy number neutral loss, such as uniparental disomy provide the genetic basis of the disease [102]. The complexity of cytogenetic abnormalities is related to the clinical severity of the disease: the more complex cytogenetic alteration is associated with poorer disease outcomes. Complex abnormalities can be further divided by the presence or absence of *TP53* mutation/alteration. Acquired somatic molecular mutations are seen in 80–90% of MDS patients, which can be grouped by gene function as follows [102]:

(1) Epigenetic regulators and chromatin remodeling factors: methylcytosine dioxygenases of the ten eleven translocated family (*TET2*), additional sex comb-like genes (*ASXL1*), DNA-methyltransferase family (*DNMT3A*), isocitrate dehydrogenases (IDH 1/2), enhancer of zeste homolog 2 (*EZH2*)
(2) mRNA splicing factors: *SF3B1*, *SRSF2*, *U2AF1*
(3) Transcriptional factors: *RUNX1*, *TP53*
(4) Signaling molecules: *NRAS*, *KRAS*
(5) Cohesin complex: *STAG2*

The most common mutations present in more than 5–10% of the patients include *TET2, SF3B1, ASXL1, SRSF2, DNMT3A, TP53, EZH2, IDH1/2, NRAS, BCOR,* and *RUNX1*; and approximately further 30 genes are altered in about 1% of patients. Most of these mutations carry a poor prognosis, except for *SF3B1*. It is to note that some elderly (70–80 years old) people carry one or more of these mutations, typically *DNMT3A*, *TET2*, or *ASXL1*, and less often *JAK2*, *PPM1D*, *SF3B1*, *SRSF2,* or *TP53*, with no hematologic malignancy, and the term “clonal hematopoiesis of indeterminate potential” was established to describe this condition [6,102,110].

#### 3.2.4. Genetic Alterations in De Novo AML

Besides the mutations mentioned above in the context of MDS and post MDS AML, some additional mutations and chromosomal rearrangements have been described in the pathogenesis of AML [111,112]. These are often classified into two broad classes of mutations: Class I mutations that confer proliferative and survival advantages and Class II mutations affecting cell differentiation and apoptosis. Recent studies, however, have identified further mutations that do not conform to any of the two classes, many promoting epigenetic modifications (Table 1). There is a collaboration between the three classes of gene alterations, which is associated with the appearance of AML phenotype [6,113,114,115,116].

#### 3.2.5. MDS, AML and Mitochondrial Metabolism

Metabolic changes are numerous in AML and have been reviewed in excellent recent articles from Barbato and co-workers and Panuzzo and co-workers [1,117]. Here we are going to discuss these metabolic changes in relation to genetic alterations in AML and MDS. In short, in AML, increased *SIRT3*-mediated OXPHOS has been described along with increased mitochondrial fitness and ROS levels, leading to drug resistance. In addition, PDH and α-KGD inhibitors, IACS-010759 and Venolex, could re-sensitize AML cells to chemotherapy [1]. Also, a previous study showed that 8% of AML patients had mutations in their ETC complex genes, with a majority affecting Complex IV [117].

#### 3.2.6. MDS, AML and Mitochondrial Dynamics, Mitochondrial Transfer

Overexpression of mitochondrial fission receptor Fis1-mediated mitophagy was found to be essential for AML cell proliferation and differentiation in vitro. In the same model, depleting Fis1 via shRNA-mediated *FIS1* knock-down resulted in cell cycle arrest, loss of self-renewal and attenuated myeloid cell differentiation [118]. In addition, increased Drp1-dependent mitophagy has been implicated in the mechanism of *FLT3*-internal tandem duplication (ITD) inhibition therapy in AML [119]. In a previous study, AML patients with high *FIS1* expression likely to be chemotherapy-resistant and were more frequently M0/M1 FAB subtypes [77]. Similarly, mitochondrial fragmentation and increased Drp1 is also associated with MDS with *CBL* exon deletion and *RUNX1* mutation, promoting dysplasia and impaired granulopoiesis [120]. Additionally, Drp-1-dependent mitochondrial fragmentation is seen in the mesenchymal stromal cells in MDS with iron overload, contributing to cell damage [121]. Interestingly, bone marrow stromal cells can transfer mitochondria to AML cells [122,123].

#### 3.2.7. Genes Discussed

Here we aim to summarize the relationship between different genetic mutations, mitochondrial metabolism, mitochondrial dynamics, and the development of MDS/AML in selected genes. It is to note that there is a significant overlap in genetic alterations in these two diseases and that some genetic alterations are also present in other hematologic malignancies.

### 3.3. Epigenetic Regulators

**Figure 4:**
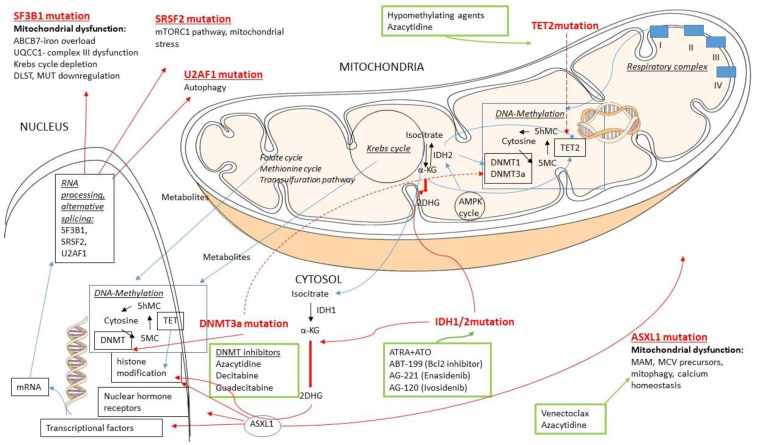
The interaction between mitochondria, epigenetic regulator and transcriptional factor mutations in hematologic malignancies. Blue arrows: connections in physiological function, Red arrows: effect of mutations, red dashed arrow: possible or not yet fully proved effect of mutation, green boxes/arrows: possible therapies. Abbreviations: DNMT: DNA-methyltransferases, TET: Ten eleven translocation enzymes, IDH: isocitrate dehydrogenases, α-KG: α-ketoglutarate, 5-MC: 5-methylcytosine, 5-hMC: 5-hydroxymethylcytosine.

Epigenetic regulation or modification means different mechanisms that alter gene expression without alterations in nucleotide sequence. The two main mechanisms of epigenetic regulation are DNA methylation and histone modifications. The interaction between DNAm, mitochondria, and transcriptional factors in hematologic malignancies are summarized in Figure 4.

#### 3.3.1. DNA Methylation (DNAm)

*DNAm mechanisms.* DNAm entails the conversion of cytosine to 5-methylcytosine (5mC) to the 5th carbon of the ring of cytosine, predominantly due to the DNA methyltransferase (DNMT) enzyme transferring a methyl group from S-adenosylmethionine (SAM). This conversion is usually found within CpG dinucleotide sites, regions of the DNA in which a cytosine nucleotide is immediately followed by a guanine nucleotide in a linear sequence in the 5′ to 3′ direction [124]. TET enzymes catalyze the conversion of 5-methylcytosine (5-mC) to 5-hydroxymethylcytosine (5-hmC) and promote DNA demethylation. Besides DNA demethylation, it also plays a role in histone modification: the mechanism that may account for TET-mediated gene activation is the recruitment of O-linked β-D-N-acetylglucosamine (O-GlcNAc) transferase (OGT) to chromatin [125]. *DNAm and related genes.* Such epigenetic DNA modifications occur in intronic, exonic and intergenic regions [126]. They are involved in the regulation of gene expression, either via interaction with promoters, enhancers, transcription factors, and gene bodies, or via stimulating transcriptional elongation and gene splicing [126,127]. With age, genome-wide methylation levels are reported to generally decrease overall across multiple tissues, referred to as hypomethylation [128,129]. This hypomethylation is more pronounced in certain tissues, such as blood and brain [130], particularly in repetitive elements of intergenic regions [131]. Dysregulated DNA methylation through mutations in DNA-methyltransferase family (*DNMT3A*)*,* isocitrate dehydrogenase *1/2 (IDH1/IDH2*), and ten-eleven translocation superfamily 2 (*TET2*) play an important role in the pathogenesis of a large proportion of MDSs and AMLs, with possible common targeted therapies in the future [110].

*DNAm genes and mitochondrial genomics.* Cells harboring distinct mitochondrial haplogroups, meaning that they possess identical nuclei with different mtDNA or mtDNA polymorphisms (such as peripheral blood, articular cartilage, and human retinal cell cybrids), have been shown to have different nuclear DNA (nDNA) methylation patterns [132,133,134,135,136]. Moreover, as many metabolites are produced in the mitochondria but transit to the nucleus, alterations in their levels can influence the efficiency of methylation enzymes and affect the production of substrates required for methylation [137,138]. These include but are not restricted to serine biosynthesis, the folate cycle, the methionine cycle, the transsulfuration pathway, and the TCA/Krebs cycle [132,139,140]. Some of the produced metabolites influence the activity of TET enzymes [137]: succinate and fumarate, as seen in multiple human cancer cell lines, are inhibitors of TET enzymes, and in the case of fumarate, it is capable of modulating TET via reducing mRNA expression of TET1 and TET2, though increasing mRNA expression of TET3 enzymes [138]. Additionally, the activity of TET enzymes also changes in response to adenosine monophosphate-activated protein kinase (AMPK)-mediated phosphorylation. AMPK can enhance the expression of TET enzymes directly or via increased IDH2 expression, a mitochondrial enzyme found in the TCA/Krebs cycle involved in the production of α-ketoglutarate (α-KG), and hence activate TETs to decrease DNAm ultimately. Nuclear DNAm can impact the expression level of nuclear-encoded genes, including nuclear-encoded mitochondrial genes, such as genes translated into proteins and enzymes required for mitochondrial transcription and replication, and structural proteins or proteins of the mitochondrial respiratory chain complex [141]. Additionally, alterations in DNAm can also be found in OXPHOS genes associated with various diseases and aging [142].

*The methylation status of the mtDNA.* For a long time after the 1970s it was a debated whether methylation occurs in the mtDNA or not [143,144,145] Using different methods and cell types, mtDNA methylation was found to range between 1 and 20%. Many authors highlighted that using the current, standard techniques of measuring DNAm, results in very low mtDNA methylation detectability and occurs differently than nDNA methylation. Interestingly, DNMTs and TETs, typically found in the nucleus, have been spotted in the mitochondria, affecting mtDNA methylation [142,146].

*Mitochondrial respiratory complex dysfunction and DNAm.* Notable data are available from studies, where rotenone-induced complex I dysfunction resulted in global changes in DNAm levels in rats, human cybrid cells, and the change was also seen in the offspring of a mouse model [147,148,149,150]. Together, these studies enforce that mtDNA alterations can be signaled to the nucleus, affecting the nDNA, and are consequently associated with nDNA methylation [132,151].

#### 3.3.2. Histone Acetylation/Deacetylation

*Histone.* Both DNAm and histone modifications, such as deacetylation, acetylation, and methylation are associated with regulation of chromatin structure and gene expression. Histone deacetylases (HDAC), which remove acetyl groups from the histones, result in more closed chromatin structure, inhibiting or decreasing gene transcription. HDAC enzymes have been found over-expressed in various malignancies, including AML. However, HDAC inhibitors have limited efficacy as single agents in some studies [110,152,153]. Interestingly, the HDAC inhibitor suberoylanilide hydroxamic acid (SAHA, Vorinostat), was found to induce PGC-1α-mediated mitochondrial biogenesis in an in vitro model of cardiac ischemia [154]. The mtDNA, as previously mentioned, lacks histones.

#### 3.3.3. Isocitrate Dehydrogenases (IDH)

*IDH.* The human genome has five *IDH* genes that encode three distinct IDH enzymes, whose activities are either NADP^+^ (IDH1 and IDH2) or NAD^+^ (IDH3) dependent. In MDS/AML, only *IDH1* and *2* have a pathogenetic role. IDH1 is cytoplasmic, whereas IDH2 is a mitochondrial enzyme. The two enzymes’ main biological functions are related to the biosynthesis of essential metabolites of the TCA/Krebs cycle that, in connection with the pentose phosphate pathway, is mainly responsible for the generation of NADPH, the main component of the cellular redox homeostasis [155].

**Figure 5:**
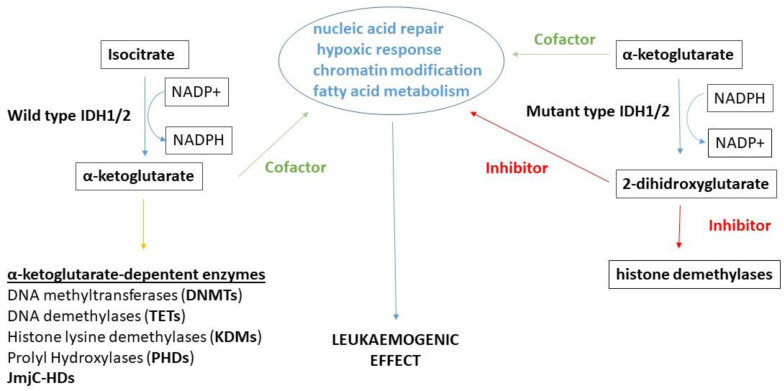
The pathogenetic role of IDH1/2. Blue and yellow arrows, black text: biochemical processes. Green text and arrows: cofactors. Red lines and arrows: inhibition.*IDH mutations in MDS and MDS/AML.* The frequency of *IDH1/2* mutations has been reported between 4 and 12% in MDS patients [156,157,158,159]. *IDH2* mutations were particularly enriched in the RAEB subtype of MDS and were mutually exclusive with *TET2* and *SF3B1* mutations but were frequently associated with *SRSF2* mutations. Many authors found that *IDH1/2* mutations were associated with poor prognosis, particularly in low-risk MDS [160,161]. The proportion of *IDH2* mutation, however, was higher than *IDH1* mutation in high-risk MDS. Both *IDH1* and *IDH2* mutations showed a significantly shorter progression time to AML when associated with low-risk mutations, such as *GATA2*, *NRAS*, *KRAS*, *RUNX1*, *STAG2*, and *ASXL1*. Another study found that only *IDH1* but not *IDH2* mutations are associated with shortened leukemia-free survival [162]. In addition, the presence of certain subtypes of *IDH2* mutations (for example *IDH2*-R172) is a predictor of poor response to chemotherapy [163]. Both *IDH1* and *IDH2* mutations are found in AMLs, with lower frequency in the pediatric patients than in the adult ones, the latter being about 20%. In therapy-related AML (t-AML), its frequency is around 7%, with *IDH1* mutations being more frequent [159,164,165,166,167,168].

*IDH and mitochondria and the pathogenetic role of IDH2 mutation.* IDHs contribute to mitochondrial metabolism, although they have an indirect but crucial effect on DNA methylation via influencing enzyme activities through the products of their activity. In mitochondria, the reductive carboxylation of α-ketoglutarate to isocitrate by IDH2 consumes mitochondrial NADPH, with citrate/isocitrate transported to the cytoplasm, where these metabolites can be oxidized to produce cytosolic NADPH. The reversed process can be used to produce mitochondrial NADPH. Mutant *IDH2*, on the other hand, catalyzes the reductive conversion of α-KG to 2-dihydroxyglutarate with concomitant oxidation of NADPH to NADP^+^. This oxidative microenvironment plays a crucial role in the altered metabolism of clonal hematopoietic cells. It is suggested that 2-dihydroxyglutarate (2-DHG) could represent the oncogenic mediator in leukaemogenetic processes. α-KG is a cofactor of many deoxygenases involved in regulating key biological processes, such as nucleic acid repair, hypoxic response, chromatin modification, and fatty acid metabolism. In contrast, 2-DHG has the opposite effect as it acts as an inhibitor of these processes. 2-DHG is a competitive inhibitor of histone demethylases, and it has an influence on hypoxia-inducible factor (HIF) prolyl hydroxylase (Figure 5) [41,155].

*The mechanism of IDH-induced leukemogenesis. IDH* mutants exert their pro-oncogenic effect by interfering with the differentiation program of hematopoietic cells. Cell culture studies showed that the expression of an *IDH*-mutant enzyme induced an increase in stem cell markers and impaired myeloid cell differentiation [169]. Recently a knock-in mouse model was established, in which the *IDH1-*R132H mutation was inserted into the murine *IDH1* locus and expressed in myeloid cells. These mutant mice displayed an increased number of early hematopoietic cell progenitors, impaired myeloid cell differentiation, anemia, splenomegaly and extramedullary hematopoiesis. The hematopoietic cells of these animals displayed hypermethylated histones and changes to DNAm, similar to those observed in IDH-mutant AMLs [170].

*IDH mutations as therapeutic targets. IDH1* and *IDH2*-mutant mouse and human leukemia models suggest that they are sensitive to all-trans retinoic acid (ATRA) and the proapoptotic effect of arsenic trioxide (ATO) by themselves or in combination with each other [170]. Besides ATRA, *IDH* mutations can act as therapeutic targets for bcl-2 inhibitors. *IDH1* and *2*-mutant primary human AML cells were more sensitive than *IDH1/IDH2*-WT AML cells to ABT-199, a specific BCL-2 inhibitor, by inhibiting cytochrome c oxidase in the mitochondrial ETC [171]. In addition, IDH-inhibitors have been proven to be valuable in models when using IDH-mutant cells by inhibiting the development of the leukemic phenotype, by decreasing the production of oncogenic metabolites responsible for inhibiting cell differentiation and changes in gene expression. These inhibitors include the mutant IDH2 inhibitor AG-221 (enasidenib), and mutant IDH1 inhibitorAG-120 (ivosidenib), which have been extensively investigated for the treatment of patients with AML or MDS with a susceptible IDH mutation [142].

*IDH2 inhibition.* At the cellular level, the main effect of enasidenib is to induce cell differentiation without apoptosis induction. In addition, it reduces the 2-DHG level markedly. Co-occurrence of mutations at the MAPK and RAS pathways level was associated with reduced clinical response to enasidenib. Furthermore, enasidenib improved outcomes in an in vivo human AML xenograft model [155]. These observations strongly supported the clinical use of enasidenib. Several preclinical trials proved the efficacy of IDH2 inhibition in combination therapy. A study using a mouse model with combined mutation of *TET2, FLT3-ITD*, and *IDH2*-R140Q; thus, triple-transformed leukemia, showed tumor sensitivity both to 5-azacytidine and to IDH2 inhibitor enasidenib. The combined treatment with these two drugs resulted in a marked potentiation of the antileukemic effect, with a pronounced decrease of leukemic blasts and with their differentiation and, particularly, with a decrease of mutant allele burden and progressive recovery of normal hematopoiesis from non-mutant stem-progenitor cells [155,172].

*IDH2 inhibition in clinical trials.* Both monotherapy with enasidenib and combination therapy with enasidenib and 5-azacytidine vs. 5-azacytidine alone showed high response rates in *IDH2* mutant AML patients [158,173,174,175]. In addition, enasidenib is a promising treatment option for relapsed/refractory MDS patients harboring *IDH2* mutation following allogenic stem cell transplantation, who had overall good response rate and an improved median of survival [176]. Efficacy and tolerability of enasidenib alone and combined with azacytidine were examined in another trial, involving patients with high-risk *IDH2*-mutated MDS: the best result, which was a 100% response, was achieved in hypomethylating-agent naive patients. Interestingly, the clearance of *IDH2* mutation was observed in some patients [177].

*IDH2 and mitochondrial dynamics.* Besides its already complex role in cellular metabolism, loss of IDH2 was associated with increased mitochondrial motility and fission, the latter via Drp1 activation and expression in in-vitro and in vivo tumor models. These changes resulted in greater tumor cell movements. The exaggerated mitochondrial trafficking was induced by ROS and subsequent hypoxia-inducible factor-1α stabilization [178]. Targeting mitochondrial fission and trafficking could be valuable therapeutic options, just as altered metabolism in tumors with *IDH2*-mutations.

#### 3.3.4. Ten-Eleven Translocation (TET2) Enzyme

*The TET enzyme superfamily.* The three enzymes of the TET family (TET1, TET2 and TET3) identified in humans are evolutionarily conserved dioxygenases. Different TET enzymes exhibit distinct expression patterns in vivo, with TET1 being mainly expressed in embryonic stem cells. TET2 and TET3 are more ubiquitous, with TET2 expression predominating in various differentiated tissues, especially in hematopoietic and neuronal lineages [179].

*The physiological role of TET2 and its contribution in hematologic malignancies.* TET enzymes are one of the homeostatic links between intracellular metabolism and epigenetic gene regulation [180]. TET dioxygenases require α-KG, oxygen and Fe(II) for their activity, which is enhanced in the presence of ascorbic acid [181,182]. As mentioned above, mutant cytosolic IDH1 or mutant mitochondrial IDH2 produce 2-dihydroxyglutarate, an ‘oncometabolite’ that inhibits 2-oxoglutarate-dependent enzymes, including TET dioxygenases. TET enzymes may also be sensitive to changes in oxygen availability and susceptible to reactive oxygen species and carcinogenic metals that displace iron such as arsenic, nickel, or chromium [183].

*TET isoforms in mitochondria.* Despite unknown mechanisms of translocation to the mitochondria, TET1 and TET2 enzymes have been found to be present in mouse neuronal mitochondria, for example in the cerebellum and Purkinje cells of aged animals [184]. Moreover, the expression of TET2 and TET3, once increased, are associated with increased levels of 5hmC (5-hydroxymethylcytosine) found both in the nDNA and mtDNA [184,185]. TET enzymes could have a role in mtDNA demethylation, with the exact functioning of such enzymes yet to be elucidated. It is still debated whether potential DNMT and TET-modulated mtDNA methylation/demethylation could influence the expression levels of mitochondrial-encoded genes and their respective functionalities or whether this phenomenon loops back into affecting the nDNA.

*Animal models.* Several studies have examined TET2 inactivation in mice: its deletion leads to hematopoietic defects, including enhanced HSC self-renewal and myeloid expansion, correlating with global loss of 5-hmC in primitive hematopoietic populations [186,187,188].

*Factors influencing TET2 function.* It was recently described that restoring TET function via inducible shRNA model of TET-induced AML, or through vitamin C administration, the latter being a cofactor for α-KG dependent dioxygenases reverses leukemogenicity induced by the mutant TET protein [189]. These results imply that metabolic control of TET activity could be harnessed for therapeutic benefit in patients with TET mutations. Notably, cytosine methylation signatures of *TET2*-mutated AML show significant overlaps with those found in *IDH1/IDH2* mutated patients. It is to note that *IDH1/IDH2* and *TET2* mutations are mutually exclusive in AML [169] but signal a common mechanism of leukemogenesis based on aberrant DNA methylation. Besides vitamin C, proteolytic processes and micro-RNA miR22 regulate TET2 function [190,191].

*TET2 and hematopoiesis*. TET2 has pleiotropic roles in hematopoiesis, including stem-cell self-renewal, lineage commitment, and terminal differentiation of specific lineages. The *TET2* gene is highly expressed in HSCs and progenitor cells and is downregulated with differentiation. Several studies on mouse models proved that TET2 acts as a tumor suppressor as well [187,188,192,193].

*The role of TET mutations in MDS and AML*. *TET2* alteration was one of the most prevalent genetic abnormalities (25–35%) identified in MDS [194,195,196,197]. A more extensive series failed to identify a strong association of TET2 alteration with clinical phenotype, risk scores or overall survival [194]. Nevertheless, in higher-risk MDS and AML with low blast count, the *TET2* status can be associated with a better response to the demethylating agent azacitidine [196]. The prevalence of *TET2* mutations is higher in secondary than in de novo AMLs. In addition, *TET2* genetic alterations could be associated with adverse outcomes in cytogenetically defined subgroups of AML patients [198,199,200,201].

*TET2 mutations in other hematologic and non-hematologic malignancies.* Somatic alterations in *TET2*, including deletions and missense, nonsense and frameshift mutations, have been identified in 10–26% of MPN patients [202,203]. Acquired somatic alterations in *TET2* were identified in 2–20% of classical MPNs, including polycythemia vera, essential thrombocytosis and primary myelofibrosis [197,204,205]. These mutations, which can be an early genetic event in the course of MPN, have no clear prognostic impact; they do not increase the risk of leukemic transformation. In some cases, however, *TET2* mutations are only seen when the disease progresses to acute leukemia [206,207]. In chronic myeloid leukemia, *TET2* mutations were associated with acute blastic transformation [208]. Additionally, *TET2* mutations were identified in 20% of mastocytosis, mostly in aggressive forms of the disease [209,210]. More recently, *TET2* mutations have been also found in 30% of patients with blastic plasmacytoid dendritic cell neoplasms [181,211]. *TET2* mutations have been also identified in mature B-cell (2%) and T-cell (11.9%) lymphomas [212,213], in 33% of angioimmunoblastic T-cell lymphomas [212], in mantle cell lymphomas [214], and diffuse large B-cell lymphomas. The latter two are also associated with an altered DNA gene methylation pattern on genes involved in hematopoietic development [215]. Interestingly, in angioimmunoblastic T-cell lymphomas, *TET2* mutations are frequently associated with *DNA methyltransferase 3A* mutations [216], and *IDH2* mutations [217]. *TET2* mutations have also been detected in a small number of solid tumors, such as in prostate cancers [218].

*TET2 in AML/MDS therapy. TET2* gene alterations were anticipated to predict an increased response to hypomethylating agents and this predictive effect was explored in several cohorts of high-risk MDS, AML with low blast counts, and severe CMML patients [219,220,221,222,223]. In some of these studies, the TET2 status appeared as an independent genetic predictor of azacitidine response, although not conferring a survival advantage [196,221].

#### 3.3.5. DNMT3 Enzyme

*The DNMT enzyme family.* The *DNMT* family, including *DNMT1*, *DNMT3A*, and *DNMT3B* encode methyltransferases that catalyze DNA methylation that involves adding a methyl group to the carbon-5 position of cytosine in CpG dinucleotides, leading to the formation of 5-methylcytosine (5-mC). DNMT3A and DNMT3B are involved in de novo DNA methylation, whereas DNMT1 plays a role in the maintenance of DNA methylation [224]. DNMT3A is highly expressed in T-lymphocytes and neutrophils, while DNMT3B is downregulated in hematopoietic differentiation. Aberrant CpG island promoter methylation in tumor suppressor genes is an important pathogenetic mechanism in malignant tumors, suggesting that DNMTs play important roles in oncogenesis [225].

*DNMT isoforms in the mitochondria.* DNMT1 has been observed to translocate into the mitochondria and interact with the mtDNA in the matrix of some tissues, such as mouse embryonic fibroblasts, human colon cancer cells [141], and human neurons [226]. The presence of DNMT3A has also been described in mitochondria. Interestingly, a particular isoform, the DMNT1-3A, was the one that has been identified to be able to both transfer to mitochondria and methylate the mtDNA [227,228,229].

*DNMT3A and leukemogenesis.* The mechanism of leukemogenesis by *DNMT3A* is not entirely clear; however, studies have shown that heterozygous *DNMT3A* ablation in mice leads to an expansion of the hematopoietic stem cell pool [230]. The formation of myeloid malignancies, however, may require additional genetic alterations. This reinforces the notion that *DNMT3A* mutation, just as mutations in other epigenetic regulators, do not necessarily lead to frank leukemic transformation on their own but rather create a premalignant state that lays the ground for malignancy. It has recently been reported that mutant *DNMT3A* (R882H) interacts with the Polycomb repressive complex 1 (*PRC1*) in order to silence genes, suggesting that PRC1 activity could be an attractive target in *DNMT3A*-mutant tumors [231].

*The role of DNMT3A mutations in MDS and AML. DNMT3A* mutations occur in 30–35% of AMLs with normal karyotype, 10% of MDSs, and 20% of T-lineage acute lymphoblastic leukemia [232,233,234]. As mentioned above, *DNMT3A* mutations result in loss of function, and can be present in pre-leukemic hematopoietic stem cells, which can remain in the tumor cells after transformation to MDS or AML [235,236]. *DNMT3A* mutations are associated with poor prognosis and decreased overall survival [237]. MDS patients with *DNMT3A* mutations have a shorter OS and higher risks of leukemic transformation [238,239]. Additionally, *DNMT3A* mutations have been observed in non-leukemic T-cells from AML patients as well and in normal elderly individuals with no signs of leukemia [240].

*DNMT3a in MDS/AML therapy. DNMT3A* mutations are associated with a positive response to DNA methyltransferase inhibitors (a.k.a. DNA demethylating agents), namely azacitidine, decitabine and guadecitabine [233,241]. The effect of histone deacethylase (HDAC) inhibitors, such as pracinostat, vorinostat and valproic acid, have been modest in AML. In combination with DNA demethylating agents, however, HDAC inhibitors show increased activity [152].

#### 3.3.6. Additional Sex Like Comb Protein 1 (ASXL1)

*ASXL superfamily.* The *ASXL* superfamily of genes and the encoded proteins consist of three members, namely *ASXL1*, *ASXL2* and *ASXL3*. They are human homologs of the *Drosophila* Asx gene, encoding epigenetic scaffolding proteins that are involved in the regulation or recruitment of the polycomb-group repressor complex (PRC) and trithorax-group (trxG) activator complex, participating in epigenetic regulation and histone modification [242]. *ASXL1* directly interacts with various protein-coding genes, such as *BAP1*, *KDM1A (LSD1)*, *NCOA1*, and nuclear hormone receptors, such as retinoic acid receptors, estrogen- and androgen receptors. The loss of the *ASXL1* gene is associated with leukemoid transformation and increased self-renewal in hematopoietic cells [242].

*The role of ASXL1 in mitochondrial and endoplasmic reticulum function.* A recent publication reported a significant mitochondria–endoplasmic reticulum deficiency in a leukemia cell line carrying *ASXL1* mutation. This deficiency was related to decreased number of matrix granules, mitochondria-associated endoplasmic reticulum membrane (MAM), and mitochondrial-derived vesicle (MDV) precursors, which are implicated in the regulation of cell death pathways via mitophagy and intracellular Ca^2+^ concentration changes. These cells are also thought to have increased mitochondrial respiration and defective mitophagy [243].

*ASXL1 mutations in hematologic malignancies.* Nonsense point mutations or frame-shift mutations of *ASXL1* occur in hematological malignancies, such as MDS, MPNs, MDS/MPNs, AMLs and CLL [244,245,246]. Whole-exome sequencing analyses revealed a 2.9% *ASXL1* is mutation rate in CLL [246], with generally higher percentages in myeloid neoplasms (45.3% in CMML, 34.5% in MPN, 30% in secondary AML, 16.2% in MDS, and 6.5% in de novo AMLs) [247]. In MDS, *ASXL1* mutations are independent adverse prognostic factors both in overall and leukemia-free survival. In addition, *ASXL1* mutations are associated with shorter overall survival in CMML patients [194,220,247,248,249]. In AML, *ASXL1* mutations were more frequently found in male [250,251,252,253,254], and older age patients [255], and in patients with lower platelet count and hemoglobin level. Of note, *ASXL1* mutations are frequently seen with other gene alterations, such as with *EZH2 IDH1/2*, *RUNX1*, and *TET2* [194,256], most of which are adverse prognostic factors themselves in myeloid neoplasms, potentially explaining why *ASXL1* mutations are associated with poor prognosis in many cases [257].

*ASXL1 in MDS and AML therapy.* Leukemic cells with *ASXL1* mutation have been shown to overexpress anti-apoptotic Bcl-2 and have increased global cytosine methylation levels. It is not surprising that these cells were sensitive to both the Bcl-2 inhibitor venetoclax and the DNMT-inhibitor azacytidine [258]. In addition, clinical trials have shown the efficacy of venetoclax combined with hypomethylating agents or low-dose cytarabine in a subgroup of AML patients [259].

#### 3.3.7. Enhancer of Zeste Homolog 2 (EZH2)

*EZH2 and carcinogenesis.* Enhancer of zeste homolog 2 (EZH2) is an epigenetic modulator, part of the polycomb repressive complex 2 (PRC2) that can suppress gene expression via histone 3 (H3K27me3) di- or trimethylation. In addition, EZH2 methylates non-histone proteins, such as the transcription factor GATA4, and can activate downstream genes in a PRC2-independent manner, contributing to its complex effect on cells. Mutation or altered expression of *EZH2* has been linked to various tumors, including hematologic malignancies and solid tumors, such as breast cancer, esophageal cancer, gastric cancer, and anaplastic thyroid carcinoma. Interestingly, both increased and decreased EZH2/PRC2 functions are associated with carcinogenesis. With increased function, EZH2 silences genes that promote differentiation and restrain proliferation. As a tumor suppressor, loss of EZH2 accelerates Ras-driven neoplastic processes and can amplify Akt and ERK activation. In *JAK2*-V617F transgene mice with concurrent EZH2 knockout, a synergistic effect with very high platelet and neutrophil count, accelerated myelofibrosis, and reduced survival was described. Additionally, PRC2 is suppressed in T-cell acute lymphoblastic leukemia (T-ALL) by NOTCH1 signaling, resulting in loss of H3K27me3 repression as part of leukemogenesis. In CML, EZH2 overexpression is induced by BCR-ABL1 via signal transducer and activator of transcription (STAT) 5 phosphorylation [260,261,262,263,264,265].

*EZH2 in malignancies.* The type of EZH2 alteration is different in different tumor types. Solid tumors often overexpress EZH2, whereas hematologic neoplasms show a more diverse picture of how *EZH2* is altered. Gain-of-function mutations, promoting H3K27me3 and subsequent gene suppression, have been described in germinal center-type diffuse large B-cell lymphomas (30%) and follicular lymphomas (20%). Gain of copy-number, as part of gains of chromosome 7 (including the 7q36.1 region), has also been described in follicular lymphomas, resulting in the same overall effect as the gain-of-function mutations in these patients. In patients with various B-cell lymphomas, high-risk MDS, and AML, overexpression of *EZH2* is the most common. Interestingly, loss-of-function mutations have also been described in MDS (6%), MDS/MPN (10–12%), and myelofibrosis (13%). [117,263,264,266]. Loss of chromosome 7, or deletion of 7q (7q-), which are frequently seen in AML and MDS patients, also involves *EZH2* [264]. In general, *EZH2* mutations are associated with inferior overall survival in myeloid malignancies, with no increased rate of AML transformation in MDS patients [246,267]. In addition, decreased EZH2 expression is associated with worse overall survival in AML [268]. Also, increased EZH2 expression was found in AML patients with complete remission when compared to newly diagnosed patients, who had no *EZH2* mutations in either of the mutated hotspots [269]. Loss of EZH2 function can either attenuate and promote leukemic transformation in MDS and MPN, depending on the disease context and cooperating mutations [268].

*EZH2 and mitochondria. EZH2* alters apoptotic pathways, with no data on its effect on mitochondrial dynamics. In glioma cells, its downregulation results in apoptosis and cell cycle arrest in the G0/G1 phase via cytochrome c release [270]. Similarly, in multiple myeloma cells, inhibition of EZH2 showed caspase-3-dependent apoptosis [271]. On the other hand, in EZH2 overexpressing melanoma cells, the EZH2 inhibitor GSK126 caused caspase-independent apoptosis, mediated by apoptosis-inducing factor, mitochondrion associated 1 (AIFM1) protein [272]. In contrast, EZH2 inhibition enhanced cell proliferation and reduced apoptosis in AML cells, in line with myeloid cell leukemogenesis and chemotherapy resistance with reduced EZH2 expression or function [269]. Nevertheless, EZH2 inhibition reduced glycolysis, promoted OXPHOS in pancreatic cells in vitro [273], and inhibited glycolysis in prostate cancer cells [274]. Moreover, EZH2 regulates lipid metabolism through the EZH2-TERT-lipid metabolism network, with EZH2 knockdown glioblastoma cells showing reduced fatty acid synthase expression and resultant diminished fatty acid levels [263,275].

*EZH2 as targeted therapy.* Patients with a gain of function mutations, or increased copy-number have been proposed to be ideal candidates for EZH2 inhibitor treatment. EZH2 inhibitors have been tested in clinical trials in various malignancies, including myeloid and lymphoid neoplasms, listed in more detail in the review of Duan and co-workers [263,266], with the existing pre-clinical data supportive of treatment in these diseases [276,277].

### 3.4. RNA Splicing Gene Mutations

#### 3.4.1. RNA Splicing

RNA splicing or alternative splicing is where mature RNA is formed from pre-messenger RNA (pre-mRNA) through intron removal and exon splicing. Alternative splicing increases the transcriptomic and proteomic complexity by generating distinct RNA isoforms with different functions than the other products from the same gene. Alternative splicing can contribute to carcinogenesis when dysregulated [278,279]. Mis-splicing of genes has been found in multiple malignancies, including some carcinomas, neuroblastoma, CLL and AML. Also, mutations in genes involved in RNA splicing, such as *SF3B1*, *SRSF2*, *ZRSR2* or *U2AF1/2*, occur in approximately 50% of MDSs and are currently intensively examined for their therapeutic relevance [280,281,282,283].

#### 3.4.2. Alternative Splicing

Alternative splicing is also regulated by epigenetic changes [284,285]. Histone modifications and DNA methylation can affect exon usage by controlling the elongation speed of RNA polymerase II and, consequently, the choice of splice sites. Moreover, chromatin modifications have been found to regulate the activity of alternative or cryptic transcriptional start sites (TSSs) in the genome [286]. Indeed, deregulation of alternative promoter usage has been recently identified as a common phenomenon in cancer [287]. It is, however, largely unknown how genetic changes can result in alternative promoter choices. In addition, their role in mitochondrial metabolism and epigenetic regulation is still unknown. Recently many RNA splicing factor mutations were detected as a possible contributor in MDS and AML pathogenesis. Until now, its role as a therapeutic target remained unclear in clinical settings, but preliminary data from experiments on cell lines showed promising results [225].

#### 3.4.3. SF3B1

*SF3B1* encodes a subunit of the splicing factor 3b complex. SF3B1 disrupts the usage of thousands of splice junctions, leading to altered expression of hundreds of genes [225]. *SF3B1* mutations are seen in 57–75% of patients with refractory anemia with ring sideroblasts (RARS) and 6–18% of patients with MDSs without ring sideroblasts [288,289].

*SF3B1 in mitochondrial metabolism.* The mutated *SF3B1* downregulates genes essential to various mitochondrial pathways, including *ACACA* (acetylcoenzyme A carboxylase α) and *RGL1* (ral guanine nucleotide dissociation stimulator like-1) genes. *SF3B1*-mutated RARS have abnormal splicing of the *ABCB7* gene in the mitochondria, which leads to deficiency of the ABCB7 protein, resulting in mitochondrial iron overload, reduced heme synthesis, and ineffective erythropoiesis [283,290]. *SF3B1*-mutated MDS is associated with thrombocytosis, increased ring sideroblasts, fewer cytopenias, lower blasts percentage, and are associated with a favorable prognosis [289].

*Metabolic effect of SF3B1 mutation.* A recent in vitro study showed that *SF3B1* mutated cells expressed less mitochondrial complex III gene, resulting in decreased cellular respiration and reduced citric acid cycle metabolites. In addition, further misspliced and downregulated mitochondrial metabolic enzymes, such as dihydrolipoamide S-succinyltransferase (DLST) and methylmalonyl-CoA mutase (MUT), were noted. This metabolic reprogramming bears a particular relevance in MDSs with ring sideroblasts (MDS-RS) with *SF3B1* mutation [291].

#### 3.4.4. SRSF2

*SRSF2,* encoding the serine/arginine-rich splicing factor 2, is critically involved in splice site selection, spliceosome assembly, and constitutive and alternative splicing [291]. *SRSF2* mutations are stable during disease evolution in MDS, suggesting that they may play a role in disease initiation. *SRSF2* mutations are seen in 11–15% of patients with MDS, frequently co-existing with *RUNX1*, *IDH1*, *IDH2*, and *ASXL1* mutations [283], and confer an inferior overall survival [292,293].

*SRSF2 and mitochondria.* In mammalian cells, nutrients and growth factors signal through an array of upstream proteins to regulate the mTORC1 growth control pathway. *SRSF2* gene function impacts the mTORC1 pathway, and mitochondrial stress influences mTORC1 signaling as well [294].

#### 3.4.5. U2AF1

The *U2AF1* gene encodes the U2 auxiliary factor, facilitating the binding of U2 snRNP to the pre-mRNA branch site. Recurrent mutations of the *U2AF1* gene occur in 9% of patients with MDS. The prognostic impact of *U2AF1* mutations and the exact mechanism of *U2AF1S34F* mutation confers a clonal growth advantage in MDS remain unclear [283,295]. FOXO3a activation is likely involved by increasing oxidative stress, which subsequently alters multiple processes, including apoptosis, autophagy, and immune-inflammatory responses [296].

### 3.5. Transcription Factor Mutations

The main effects of the transcription factors discussed are summarized in Table 2.

#### 3.5.1. Runt-Related Transcription Factor 1 (*RUNX1*)

All RUNX proteins contain the runt-homology domain (RHD), which is responsible for DNA-binding and interaction with a common heterodimeric partner, CBFb [297]. The fusion oncogene RUNX1/RUNX1T1 is expressed due to the chromosomal translocation t(8; 21), which involves the RUNX1 gene on chromosome 21 and the RUNX1T1 gene on chromosome 8 [298]. When expressed in hematopoietic cells, the fusion protein occupies more than 4000 genomic sites and forms transcription regulatory complexes by recruiting cofactors [299,300,301,302,303,304]. These complexes trigger local chromatin remodeling of a wide range of genes and thereby affect their expression [299,305,306]. The change of target gene expression leads to a block of cell differentiation, increased self-renewal, inhibition of apoptosis, which eventually results in malignant transformation [301,305,307,308]. The details of all these events, however, are not entirely understood.

*RUNX1 in mitochondria.* In a mouse model with both *RUNX1* mutation and CBL exon deletion increased Drp1-dependent mitochondrial fission and ROS production in the tumor cells, leading to impaired granulopoiesis dysplasia and an overall MDS phenotype. These changes were reversible with Drp1 inhibition, making it a promising therapeutic candidate in MDS patients harboring RUNX1 mutation/RUNX1/RUNX1T1 fusion gene [120].

*RUNX1 in AML.RUNX1* and *CBFB* are frequent targets of chromosome abnormalities in human AML and ALL. Core inding factor (CBF) leukemia subtypes, which includes leukemias harboring fusion genes *CBFB/MYH11* or *RUNX1/RUNX1T1*, are associated with younger age and range from 20% in pediatric to less than 5% in older AML patients [309]. Moreover, CBF leukemias are generally associated with a relatively good prognosis. Somatic mutations in *RUNX1* are detected in approximately 3% of pediatric and 15% of adult de novo AML patients. In cytogenetically normal cases, the presence of *RUNX1* mutations is associated with poor prognosis [310,311,312].

*RUNX1 in MDS.* Several studies reported somatic mutations in *RUNX1* in patients with primary MDS, therapy-related MDS (t-MDS), and AML from MDS progression [313,314,315,316,317,318,319,320]. RUNX1 mutations also occur in 20% of Fanconi anemia and 64% of congenital neutropenia (CN) patients, who later develop MDS [310,321]. *RUNX1* mutations in MDS are distributed throughout the gene, affecting both major functional domains. *RUNX1* is one of the most frequently mutated genes in MDS, accounting for roughly 10% of the cases [320,322]. In primary MDS cases, there is a positive correlation between *RUNX1* mutations and shorter survival [323].

#### 3.5.2. Tumor Protein 53 (TP53)

*TP53 and apoptosis inhibition, mutant p53. TP53* is a tumor suppressor gene encoding the tumor suppressor protein p53. When p53 forms a homotetrameric transcription factor, it directly regulates about 500 target genes, resulting in various changes, including cell cycle arrest and cell senescence, DNA repair, metabolic adaptation, and apoptosis. Of note, there is significant cross-talk between *TP53* and *MYC*. Additionally, p53 induces apoptosis and autophagy in a transcription-independent way, interacting with other proteins in the cytoplasm [324]. The induction of apoptosis by p53 involves the transcriptional activation of the pro-apoptotic proteins PUMA, and-to a lesser extent-NOXA. PUMA and NOXA inhibit the anti-apoptotic bcl-2 and bcl-Xl proteins, leading to the release of bax/bak inhibition and cytochrome-c release from the mitochondria [325]. In the absence of functional p53, the cells enter to S-phase [326]. In addition, the loss of p53 stabilizes topoisomerase IIα on the DNA and causes fork retardation, leading to torsional stress between the two machines [326,327]. *TP53* is an unusual tumor suppressor. In contrast to other tumor suppressors, such as *RB* and *PTEN*, where protein expression is significantly deceased or lost, mutant p53 expression can be high compared to the wild type (WT) p53 [325]. The mutant p53 can participate in tumorigenesis in three different ways: 1. Loss of WT p53 activity 2. Dominant negative effect of the mutant form over the WT p53, via formation of unfunctional mixed tetramers 3. Gain of function mutations, where the mutant p53 affects other transcription factors and tumors suppressors (such as p63, and p73) [325].

*TP53 and metabolism.* Although inhibiting apoptosis is an important part of TP53-related carcinogenesis, it also greatly impacts cellular metabolism and mitochondrial dynamics. This is supported by the in vivo mouse model, where the animals lacking the critical effectors of p53-apoptosis do not develop tumors spontaneously, as the TP53-deficient mice [325,328]. The transcription of glucose transporters GLUT1, GLUT3, and GLUT4 are downregulated by p53. In addition, p53 also inhibits GLUT1 translocations, and downregulates the expression of several glycolytic enzymes, such as hexokinase 2, and phosphoglycerate mutase 1. Furthermore, p53 increases parkin expression, which initiates the degradation of HIF-1α, the latter of which promotes glycolysis under normal conditions. Additionally, the p53-inducible gene TP53-induced glycolysis and apoptosis regulator (*TIGAR*) lowers the levels of reactive oxygen species (ROS) and fructose-2,6-biphosphate, thereby further inhibiting glycolysis. P53 further represses glycolysis by the transcriptional inhibition of *PFKFB3* and *PFKFB4* genes, which reduce fructose-2,6-bisphosphate expression. In addition, p53 inhibits the pentose phosphate pathway (PPP) by direct binding to glucose-6-phosphate dehydrogenase (G6PD), and it also inhibits the expression membrane-bound lactate transporter. OXPHOS is induced by p53, which represses the transcription of pyruvate dehydrogenase kinase 2 (PDK2), a negative regulator of pyruvate dehydrogenase (PDH) that converts pyruvate to acetyl-CoA, a primary substrate in the TCA cycle. OXPHOS is enhanced by p53 via the activation of mitochondrial glutaminase 2 (GLS2), catalyzing the hydrolysis of glutamine to glutamate. Additionally, p53 suppresses fatty acid synthesis and enhances lipid synthesis, unfavorable for tumor cells. Interestingly, *TP53* with a gain-of-function mutation promotes glycolysis [329,330,331]. The widespread effect of p53 on cellular metabolism explains why tumors harboring an altered *TP53* are more aggressive. These effects also represent possible therapeutic targets.

*TP53 and mitochondrial dynamics.* Supporting cellular senescence, p53 promotes the formation of highly interconnected mitochondria by blocking fission protein Drp1 translocation to the mitochondria. This translocation inhibition can occur via inhibitory phosphorylation of Drp1 at the Ser637 site. This inhibitory action is part of its tumor suppressor effect. P53-dependent Protein kinase A (PKA) activation has also been linked to mitochondrial elongation [332,333]. On the contrary, a recent study showed that mitochondrial fusion protein OPA1 is cleaved by p53 via another inner mitochondrial membrane protein, OMA1, resulting in bax/bak-dependent apoptosis induction. This pathway is suppressed in normal cells, where different stressors can activate it. OMA1/OPA1 expression is, however, variable in different solid tumors, and their exact role in cancer needs to be further investigated [334]. In patient-derived T-ALL xenografts, increased OPA1-cleavage and resultant mitochondrial fission and cell death could be triggered by ROS-generating therapy [335].

*TP53 in tumors.TP53* mutations have long been described in the literature along with Li-Fraumeni syndrome, where the patient carries a heterozygous mutation of the *TP53* genes, resulting in the development of multiple malignancies [336,337]. Most of the mutations are point mutations in the DNA-binding domain, resulting in the loss of function of the gene. Gain of function mutations, and dominant negative effect, however, have also been reported. Regarding the dominant negative effect, numerous mutant p53 would form mixed tetramers with the WT p53 in a patient with retained *TP53*, resulting in impaired function. These mutations depend on the tumor cell type, with many malignancies harboring various possible *TP53* mutations [325].

*TP53 in hematologic malignancies.* In MDS patients, *TP53* mutations, especially mutli-hit *TP53* mutations, are associated with high-risk disease, rapid transformation to AML, and therapy resistance. On the other hand, patients with monoallelic *TP53* mutations had a higher incidence of other genetic mutations, including genes, such as *TET2*, *SF3B1*, *ASXL1*, *RUNX1*, *SRSF2*, *JAK2*, *BCOR* and *CBL* [338]. In AML, *TP53* mutations occur in about 5–10% of patients, with a higher incidence in the t-AMLs, and are associated with therapy resistance and short overall survival [339,340]. In addition, AML patients with *TP53*-aneuploidy showed inferior outcomes [341]. Furthermore, *TP53* mutations are frequently seen in lymphoid malignancies, such as in DLBCL (20–25%) and mantle cell lymphoma (25%) [342,343]. Additionally, patients with secondary DLBCL harboring *TP53* mutations and treated with R-CHOP had inferior outcomes [342,343]. In B-cell lymphomas, altered *TP53* and *MYC* expressions can co-exist, with a significant cross-talk between the two pathways, accelerating each other’s effect. Given that both *TP53* and *MYC* alterations are associated with worse outcomes, their co-existence results in particularly poor prognosis [324]. In multiple myeloma, *TP53* alterations include monoallelic deletion as part of deletion of chromosome 17p (del17p) (~8%), monoallelic mutations (~6%), and biallelic inactivation (~4%), associated with high-risk disease [344].

### 3.6. Signaling Molecules

#### RAS

*RAS* proto-oncogenes, such as *HRAS*, *NRAS*, and *KRAS*, encode small GTPases and are frequently seen in various tumors. They exist in GTP-bound active, and GDP-bound inactive forms. When activated, they modulate a wide range of gene transcription. Oncogenic *RAS* mutations commonly result in amino acid substitutions, locking Ras proteins in a GTP-bound and constitutively active state [345]. Ras overexpression can cause a so-called “replication stress”, which is linked to carcinogenesis similarly to myc. Ras-induced accelerated transcription is driven by transcription factors, such as TBP, which are required for all promoters. On the other hand, c-Myc amplifies transcription indirectly by up- or downregulating target genes that enable RNA production [326]. Interestingly, Ras and Myc have a functionally connected network, and Ras pathways can regulate Myc [345,346]. The major downstream Ras effector pathways include the phosphatidylinositiol-3-kinase (PI3K), mitogen-activated protein kinase (MAPK), and Ras-like (Ral) small GPTase signaling pathways [346].

*RAS-induced metabolic changes.* Ras and Myc similarly suppress OXPHOS and enhance glycolysis, described in various tumors [13]. Ras, however, reprograms cellular metabolism more robustly than Myc [345]. The signal transducer and activator of transcription 3 (STAT3) is one of the key transcription factors regulating glycolysis and OXPHOS in a Ras-dependent manner [347]. In addition, both Ras and Myc are associated with increased ROS production [326].

*RAS-induced changes in mitochondrial dynamics.* Increased Ras expression and Erk activation leads to enhanced Drp-1-dependent mitochondrial fission, which was necessary for Ras-induced carcinogenesis in preclinical models [3,80]. Interestingly, T-ALL cells show Erk/Drp1-dependent mitochondrial fission in vitro, triggered by surrounding mesenchymal stem cells [348].

*RAS in hematologic malignancies.* Both AML and MDS frequently harbor *RAS* mutations, with *NRAS* predominating [346,349]. Although *NRAS* mutations are frequently associated with AML progression from MDS [350], several large cohort studies indicated that *NRAS* mutations in primary AML did not influence the prognosis of patients. However, a co-existent *USAF1* mutation in AML with *RAS* mutation, however, was related to chemotherapy resistance [351]. Targeting downstream pathways of accelerated Ras expression have been used in various clinical trials with variable outcome. Targeting Ras-like (Ral) GTPases, which are critical mediators of Ras-driven transformation in AML, is a further therapeutic option [346], along with targeting cellular metabolism and mitochondrial fission.

### 3.7. Myeloproliferative Neoplasms (MPNs)

As the name suggests, myeloproliferative neoplasms (MPNs) are characterized by overproduction of a specific cell line, such as white blood cells, red blood cells, and platelets. The quintessential disorder in this category of hematologic disorders is chronic myeloid leukemia (CML). However, it also includes polycythemia vera (PV), essential thrombocythemia (ET), primary myelofibrosis (PMF), chronic neutrophilic leukemia (CNL), chronic eosinophilic leukemia (not otherwise specified) (CEL), and other unclassifiable myeloproliferative neoplasms. The chromosomal abnormality associated with CML is t(9;22), resulting in the fusion of *BCR*-*ABL1* genes, also termed the Philadelphia chromosome. Although chromosomal abnormalities do not characterize most myeloproliferative disorders, other potential abnormalities include del(9p), del(13q), del(20q), gains of chromosome 8 or 9; and those more suggestive of primary myelofibrosis include der(6)t(1;6)(q21-23;p21.3). In addition, the presence of various genetic mutations are common, and include mutations in Janus kinase 2 (*JAK2*), and less frequently calreticulin (*CALR*) or thrombopoietin receptor (myeloproliferative leukemia virus gene, *MPL*). The most prevalent alteration in *JAK2* is a phenylalanine replacement of valine at position 617, commonly referred to as *JAK2*V617F. Other mutations in exon 12-15 of *JAK2* also occur to a lesser extent [6].

#### 3.7.1. BCR/ABL1, JAK2, CALR and MPL

The most common genetic alterations described above, including *BCR*/*ABL*, *JAK2*, *MPL*, and *CALR*, exert their effects through alterations in tyrosine kinase signaling. The translocation of the Abelson 1 (*ABL1*) oncogene on chromosome 9 to the Breakpoint Cluster Region (*BCR*) on chromosome 22 results in the fused *BCR*-*ABL1* gene. The following protein product induces neoplastic proliferation through deregulated tyrosine kinase activity. The combined BCR-ABL1 product loses its regulatory activity and is thus constitutively active. The unaltered ABL1 protein plays a central role in numerous processes, including cellular survival, proliferation, migration, and stress response. Therefore, loss of its regulation leads to enhanced and persistent signaling through the PI3K/AKT/mTOR, RAS/RAF/MEK/ERK, and JAK2/STAT pathways [352,353]. Likewise, alterations in the *JAK2*, *CALR*, and *MPL* genes lead to transformed tyrosine kinase activity and enhancement of similar signaling pathways. The most common mutation, *JAK2*V617F, results in loss of the JAK2 inhibitory domain, allowing for persistent downstream activation. The MPL product is the thrombopoietin receptor, which normally activates the JAK2 pathway when bound by thrombopoietin. Mutations in *MPL* also result in loss of auto-inhibition and thus autoactivation of the JAK2/STAT signaling pathways [354]. On the other hand, CALR functions as a chaperone protein to control calcium equilibrium and protein folding. Mutations in *CALR* were more recently discovered and are thought to exert oncogenic effects through its altered C terminus. The altered CALR can subsequently bind with MPL intracellularly before being trafficked to the cell surface where it can constitutively activate the thrombopoietin receptor without ligand binding [355].

#### 3.7.2. Metabolic Changes in MPNs with Altered JAK2

Numerous biological derangements are associated with the previously described mutations commonly detected in MPNs. The subsequent metabolic alterations are closely linked to mitochondrial biogenesis and include modifications in apoptosis, glycolysis, fatty acid metabolism, glutaminolysis, and subsequently in OXPHOS [356,357,358]. Ligand independent activation of the JAK/STAT pathway is a common oncogenic modality amongst MPNs. Thus, the underlying metabolic changes induced by this activation are essential for disease progression, neoplastic cell survival and proliferation. Glucose usage is dramatically increased amongst JAK2V617F expressing cells. This upregulation of metabolic processes appears to result from enhanced glucose transportation into the cell via the GLUT1 transporter and increased activity of the glycolytic rate-limiting enzyme, 6-phosphofructo-1-kinase. The enhanced activity of 6-phosphofructo-1-kinase depends on 6-phosphofructo-2-kinase/fructose-2,6-bisphosphatase 3 (PFKFB3), which is controlled by the JAK2V617F kinase. These metabolic changes enhance energy production through glycolysis and the subsequent shunting into OXPHOS within the mitochondria [358]. Not only are the metabolic processes of the neoplastic cells altered, but they also induce changes in neighboring cells by inducing stromal cells to release amino acids, lipids, and other molecules that can be used for energy production. This process contributes to disease progression and can ultimately result in cachexia through starvation of healthy cells. MPN cells also demonstrate increased lipid metabolism and pentose phosphate pathway due to hypoglycemia and inflammatory cytokines. Furthermore, glutamine metabolism is ramped up through increased glutamine uptake from surrounding cells to meet high energy demands [356].

#### 3.7.3. MPN Treatment

The treatment of many MPNs is often based on cytoreductive therapy (hydroxyurea) and the prevention of secondary complications (thrombosis) with low-dose aspirin or phlebotomy for PV. Bone marrow transplantation is also available to some patients with MPN, such as high-risk PMF. However, Janus kinase inhibitors, such as ruxolitinib and fedratinib, are also available to patients who fail or cannot tolerate initial therapy. Although rather efficacious in treatment, Janus kinase inhibitors often do not entirely eliminate abnormal myeloid progenitors, contributing to disease relapse. Therefore, these medications are marred by potential full relapse when discontinued. Current investigations have shown that deubiquitinase (DUB) inhibitors (WP1130 and G9) are potentially helpful treatment modalities to induce apoptosis in JAK2V617F mutants. DUB inhibitors can exert their effect by preventing the deubiquitination of JAK2 so that altered JAK2 molecules are passed through the ubiquitin/proteasome pathway. Additionally, DUBs are important in maintaining mitochondrial quality control, and thus their inhibition leads to amplified ROS production Moreover, DUB inhibitor WP1130 induces the activity of Bak and, to a lesser degree, Bax. Consequently, the induction of ROS and Bak/Bax leads to activation of the mitochondrial-dependent apoptotic pathway [359]. Patients with *CALR* mutations are managed similarly to most other MPNs. However, the trafficking of mutant CALR to the cell surface provides a viable potential treatment modality with either mutant CALR-binding antibody or CALR targeting by T-cell therapy [355]. Metabolism targeting drugs have also shown promise in MPN treatment, such as blocking0 of the glycolytic enzyme PFKB3 by PFK15 [356].

#### 3.7.4. Metabolic Changes Related to BCR/ABL1

Fusion of BCR/ABL1 results in similar features to those described with JAK2, and it is thought to be due to rampant cellular signaling through multiple pathways due to the kinase activity. BCR-ABL is a vital anti-apoptotic factor by activation of multiple pathways, including Ras, PI3K/AKT, and JAK/STAT pathway, in addition to Myc expression. The PI3K/AKT pathway leads to the inactivation of pro-apoptotic factors by phosphorylation, including Bcl-2 associated death promoter (Bad), forkhead box O transcription factors (*FOXO*), procaspase 9, and Yes-associated protein (*YAP*). Similarly, Ras pathway activation induces the PI3K/AKT pathway. Activation of the JAK/STAT pathway promotes cell survival by regulating Bcl-XL and Bcl-2, critical anti-apoptotic proteins [360]. Interestingly, evidence has also shown that BCR/ABL translocation can simulate hypoxic-like signaling even in the presence of oxygen. This results in the activation of hypoxia-inducible factors 1 and 2 (*HIF1*, *HIF2*), STAT5, and the glucose transporter genes *SLC2A1* and *SLC2A3*, independent of the environmental oxygen levels. STAT5 and HIF2 are important regulators of glucose uptake and utilization via the SLC2A1/3 transporters. Along the same lines, hexokinase expression is elevated, corresponding with high pyruvate levels for the TCA cycle. Furthermore, HIF1 enhances pyruvate dehydrogenase kinase (PDK2/4) so that the elevation in pyruvate can be quickly used in the TCA cycle. In addition, multiple amino acid levels were elevated compared to normal to provide additional substrates to the TCA cycle. This was partially completed through the use of the SLC1A5 glutamine transporter. Metabolism of glutamine can produce glutamate, which can further be processed into α-ketoglutarate and directly shunted into the TCA cycle within mitochondria. Glutamine appears to be an important factor for intensified OXPHOS, as treatment of BCR/ABL positive CML cell lines with a glutaminase inhibitor drastically hampered OXPHOS [361].

#### 3.7.5. CML Treatment

Due to the underlying pathogenesis of *BCR*/*ABL* fusion, treatment is often via tyrosine kinase inhibitors (TKIs), such as imatinib. Subsequent second and third-generation TKIs have also been produced in an attempt to overcome TKI resistance. However, despite their effectiveness, some CML patients develop resistance to them, possibly by altered apoptotic pathways, such as by upregulated anti-apoptotic, and pro-survival molecules, such as SRC kinases, FOXO1, XPO1, and STAT3, or by mitochondrial protein changes [352,357]. The TKI resistance is furthermore thought to occur through accumulated mtDNA damage from increased ROS production. mtDNA damage may accumulate in genes required for OXPHOS, apoptosis, and more, leading to reduced effectiveness of these pathways or even heightened production of ROS, which further damages the mtDNA. In addition, as described previously, mitochondria of CML cells have increased uptake and utilization of glucose through glycolysis and oxidative phosphorylation, which also lends a hand to the overproduction of ROS. Considering the high level of ROS detected in CML cells, oxidative damage may also play a role in a TKI resistance. Therefore, targeting mitochondrial metabolism may prove helpful in the future for TKI resistant CML patients [357].

### 3.8. Lymphomas, Acute and Chronic Lymphoid Leukemias

#### 3.8.1. Lymphomas

Lymphomas are a diverse group of lymphoid malignancies with heterogeneous molecular, chromosomal, and epigenetic changes. Lymphomas are typically divided into Hodgkin (HL) (10%) and non-Hodgkin lymphomas (90%), with the latter being either B-cell (90%), T-cell or natural killer cell (NK cell) (10%) types [362].

#### 3.8.2. Hodgkin Lymphomas (HLs)

In classical HL (cHL) (95%), there is only a small percentage of neoplastic Reed-Sternberg (RS) and Hodgkin cells intermixed with inflammatory cells. On the other hand, in the nodular lymphocyte-predominant Hodgkin lymphoma (NLP-HL) (5%), the neoplastic cells are different and are designated as lymphocyte-predominant cells, a.k.a. “popcorn” cells. The mainstream treatment of HL is typically with ABVD (doxorubicin, bleomycin, vinblastine, and dacarbazine) and irradiation, where the chemotherapy has been described to primarily increase oxidative stress [1,363,364]. In relapsing/refractory (r/r) HL, about 10% of HL cases, autologous stem cell transplantation (ASCT) has about a 50% success rate. Several HL patients, however, are not eligible for ASCT or can have a relapse post-ASCT [365]. Newer drugs, such as anti-CD30 antibody-drug conjugates and anti-PD1 checkpoint inhibitors, can be used in these patients, with some non-responsive to these treatments [365]. However, additional therapeutic options for these patients are still in need, which include drugs targeting mitochondria. A previous study showed that the cHL cells have largely increased OXPHOS with increased mitochondrial mass and biogenesis and low levels of lactate production, which is unusual for other types of cancer cells. In addition, increased mitophagy, mitochondrial turnover, and mitochondrial anti-oxidant capacity have been reported in HL, contributing to drug resistance [1,366].

#### 3.8.3. HL and Genetic Abnormalities

Genetic analysis in cHL is challenging due to the presence of less than 5% tumor cells in the tissue. A recent study, however, analyzed the tumor cells after microdissection, and found that a large proportion (87%) of the cells harbored mutations in the JAK-STAT signaling pathway, including genes *STAT3*, *STAT5B*, *JAK1*, *JAK2*, and *PTN1* [367]. Furthermore, about 10% of cHL patients had *TP53* point mutations/deletions, and about 60% had gains of *MDM2*, latter being a negative regulator of p53 [368]. In addition, chromosomal translocations involving the *BCL6* gene was detected in 48% of NLP-HLs [369,370].

#### 3.8.4. Non-Hodgkin Lymphomas (nHLs)

The by itself also heterogeneous group of B-cell nHLs includes several subtypes, where the tumor cells have a gene-expression profile reflecting their equivalent healthy cells of origin. In addition, they show recurrent genetic, epigenetic, and other molecular changes [371]. Typical translocations include t(14;18) in follicular lymphoma (FL) (involving Bcl2), t(11;14) in mantle cell lymphoma (MCL) (involving cyclin D1), t(8;14) in Burkitt lymphoma (overexpressing the *MYC* gene). T-cell and NK/T-cell lymphomas are rare, making up about 10–15% of nHLs. Molecular and chromosomal studies are rarely used in addition to immunophenotyping and T-cell receptor rearrangement studies, with the only exception being the frequently recurring chromosomal translocation t(2;5)(p23;q35), characteristic of ALK-positive anaplastic large-cell lymphomas, where the ALK-negative form has an inferior outcome. In peripheral T-cell lymphomas, alterations in p53, bcl-2, bcl-xl, CD26, EBV, CCND2, CCR4, PRDM1, and TCR gamma are associated with unfavorable prognosis [372]. Cytotoxic T-cell and monocytic/dendritic signatures are also associated with poor prognosis in T-cell lymphomas. In addition, high expression of GATA3 and TBX21 are predictors of inferior outcome in the “Peripheral T-cell lymphoma, not otherwise specified (PTCL-NOS)” subgroup. MYC and IDH2 alterations were detected in a smaller portion of the patients, which holds a great additional therapeutic potential [373].

*B-cell nHLs and genetic abnormalities.* In DLBCLs, the activated B-cell subtype (ABC) displays worse outcome compared to the germinal center B-cell subtype (GCB). Patients in the ABC type frequently carry mutations in the B-cell receptor (BCR) and the NF-kB pathway genes, such as *MYD88*, *CD79A/B*, *CARD11*, and *TNFAIP3*, and display active BCR signaling. In addition, *MYD88* mutations lead to constitutive activation of the JAK-STAT signaling pathway in these patients. On the other hand, *BCL2*, *MYC*, *EZH2*, and *PTEN* alterations are more commonly seen in patients with GCB type. Treatment sensitivity, however, are heterogenous in both groups, where *MYC*, *BCL2*, and *TP53* alterations have been related to therapy resistance. In addition, *TP53* mutations carry poor prognosis, especially when co-existing with altered *MYD88* [370,374,375]. In FL, besides altered *BCL2*, genes involved in the JAK-STAT and NF-κB signaling pathways in addition to *MYC* dysregulations are common. Alterations in *BCL2*, *TP53*, *EZH2*, *CREBBP, KMT2D,* and *MEF2B* are more often seen in patients with FL transformation to DLBLC or Burkitt lymphoma [370,376,377,378,379,380]. In Burkitt lymphoma itself, c-*MYC* deregulations, such as translocations and mutations, is highly characteristic. Besides others, *TP53*, *MDM4*, *CCND3*, and *TCF3* mutations have also been reported in Burkitt lymphoma [370]. MCLs characteristically have increased *CCND1* expression. Additionally, dysregulation in *TP53, MEF2B, NOTCH2, WHSC1,* and *BIRC3.* The mTOR pathway is often activated in MCL, which is related to therapy resistance. In addition, *TP53* mutations are related to poor prognosis and therapy resistant in MCL [370,380,381]. “Double-hit” (with MYC, *BCL2*, or *BCL6* alterations) and “triple-hit” (with MYC, *BCL2,* and *BCL6* alterations) lymphomas are also aggressive in nature, and are part of the high-grade B-cell lymphomas. Double-hit FLs, however, seem to have an indolent clinical behavior [382,383].

*nHL treatment.* nHL treatment is diverse and includes therapies from R-CHOP (rituxiban-cyclophosphamide, hydroxydaunorubicin, Oncovin, prednisone), through bone marrow transplantation, to gene transfer therapy, using chimeric antigen receptor-modified T-cell (CAR-T) therapies, and more. We will not discuss these in details, but excellent reviews, such as those from Nebrinsky and co-workers, Chung et al., Klener and Klanova, or Othman and co-workers are suggested in this topic for those interested [370,381,384,385]. In addition, therapeutic challenges for NK/T-cell lymphomas are discussed in articles, such as the ones from Xue and Zhang, or Wang and co-workers [386,387].

*nHLs and mitochondria.* Several genes involved in the development of nHL have been or will be discussed separately with their effect on mitochondrial metabolism and dynamics. Generally: In vitro studies showed that inhibiting glycolysis in lymphoma cells can induce apoptosis and may overcome multidrug resistance [388]. In DLBCL, different metabolic subgroups exist, with one being mitochondria-predominant, and the other one predominantly using fatty-acid oxidation and glutathione synthesis, latter insensitive to inhibitors of BCR signaling [389]. In addition, tumors with activated mTOR pathways may become resistant to mTOR inhibitors via upregulating glutaminase to enhance glutamine metabolism to replenish the TCA cycle, even in hypoxic conditions, which could potentially be targeted to fight therapy resistance. Tumors with *MYC* mutations for example are sensitive to glutaminase inhibition [380]. The heterogeneity of the nHL cells in vivo, however, underlines the need of individualized treatments. Lastly, in Burkitt lymphoma cells, increased citrate synthase, IDH, and decreased succinate dehydrogenase expression was described [390]. Interestingly, somatic mtDNA mutations are not significant in the pathogenesis of DLBCL [391], higher increased copies of mitochondrial DNA (mtDNA) associated with persistent disease status in nHL patients [390].

#### 3.8.5. Chronic Lymphocytic Leukemia (CLL)

CLL is the most frequent type of leukemia in adults, characterized by the clonal expansion of predominantly mature B-lymphocytes. It has a generally good prognosis. A frequent genetic lesion, the 13q14 deletion is seen in 50–60% of patients and has a favorable prognosis. Interestingly, unlike in other types of mature B-cell malignancies, translocations involving the immunoglubulin heavy chain is rare. Deletion of 11q22-q23 (del11q), involving the tumor suppressor gene ataxia telangiectasia (ATM) is associated with poor prognosis. The frequent trisomy 12 carries an immediate risk by itself, but has a poor prognosis when co-existent with *NOTCH1* mutations. In addition, Richter transformation is noted to be more common in patients with trisomy 12. Similarly, loss of *TP53* with deletion of the short arm of chromosome 17, and *TP53* mutations exhibit a poorer prognosis, and resistance to chemotherapy in CLL. Further mutations, such as *SF3B1, KRAS, NRAS, BRAF, MYD88, EGR2, MAP2K1, ATM, NOTCH1, POT1, CHD2, XPO1, BIRC3, MED12, FBXW7, ASXL1, NFKBIE, TRAF3, RPS15*, and *DDX3X* are also seen in CLL patients. *SF3B1* carries a good prognosis [392,393,394,395].

CLL and mitochondrial changes. Increased mitochondrial mass with accelerated OXPHOS, and increased ROS generation are characteristic of CLL cells. Similarly, *TP53* deletion or deficiency enhances glycolysis and increases mitochondrial mass, latter likely by enhanced PGC-1α expression in CLL cells. Both *TP53* deficiency and 17p deletion subsequently leads to altered metabolism, impaired autophagy and subsequent Ibratinib-resistance [1,396,397,398]. Nonetheless, according to one study, there is a positive association of increased mtDNA copy number and future CLL risk [399].

#### 3.8.6. Acute Lymphoblastic Leukemia (ALL)

The vast majority of ALLs are B lymphoblastic leukemias (B-ALLs), mostly occurring in children and adolescents. Inferior clinical outcome has been described in relation to the presence of *BCR-ABL1* fusion gene, *KMT2A* (MLL) rearrangements, or hypodiploidity (<44 chromosomes). The genetic mutations are diverse in B-ALL and include: (1) Transcriptional factors promoting early lymphoid cell development, such as *PAX5*, *IKZF1*, *EBF1*, *ETV6*, *ERG*, *GATA3*, and *LMO2.* (2) Tumor suppressor genes and cell cycle regulators, such as *TP53*, *RB1*, and *CDKN2A/CDKN2B, BAK1.* (3) Cytokine receptors, such as *CRLF2, RPOR* (4) Kinases, such as *ABL1*, *ABL2*, *CSF1R*, *JAK2*, *PDGFRB.* (5) Ras signaling pathway genes, such as *KRAS*, *NF1*, *NRAS*, *PTPN11.* (6) Lymphoid signaling genes, such as *BTLA*, and *CD200*, and (7) Epigenetic modifiers, such as *EZH2*, *CREBBP*, *SETD2*, *MLL2*, and *NSD2*. Amongst others, alterations in *IKZF1, TP53-, CDKN2A*, CREBBP, NR3C2, MSH6, PRPS1, PRPS2, NT5C2 are *ETV6* are associated with unfavorable outcome and/or therapy resistance in B-ALL [400,401,402,403]. Regarding mitochondrial biogenesis in B-ALL, a recent pre-clinical data showed high oxygen consumption, which can be a future therapeutic target in therapy-resistant B-ALL [404].

#### 3.8.7. MYC

The MYC family includes three proto-oncogenes: *MYC*, *MYCN*, and *MYCL*. The *MYC* gene codes the c-Myc protein, a transcription factor involved in cell growth, differentiation, metabolism, and cell death regulation [405,406]. c-Myc has been reported to indirectly increase transcription of multiple genes via activating discrete gene sets, resulting in increased global mRNA expression and turnover [326,407]. Similarly to Ras, c- Myc can activate p53, induce ROS generation, replication stress, and can cause changes in cellular metabolism [326,345,408]. In addition, it increases glycolytic activity via increasing the expression of key metabolic enzymes in the glycolytic pathway, such as glucose transporter GLUT1 and GLUT3, and hexokinases that convert pyruvate to lactate. MYC also increases lactate and glutamine transporters. OXPHOS was found to be suppressed or increased with glycolysis typically predominating [7,329,345,408,409,410,411,412]. Besides increased ATP generation, these changes can effectively increase nucleotide, amino acid and fatty acid synthesis [41,412]. In addition, MYC increases β-oxidation [413] and glutaminolysis [329]. Myc also promotes mitochondrial fission via increased Drp1 and Mff [81], and increases mitochondrial biogenesis and replication via increased *PLOG, PLOG2*, and *NRF1* expression [414]. The expression of mitochondrial trafficking proteins is also induced by c-Myc via upregulation of RHOT1, RHOT2, TRAK2, and Kif5B, resulting in enhanced organelle movements and mitochondrial recruitment to the cortical cytoskeleton [50].

Aberrant expression of c-Myc has been described in various hematologic malignancies, including Burkitt lymphoma, “double-hit/ “triple-hit” DLBCLs, high-grade B-cell lymphomas, multiple myeloma (MM), some mantle cell lymphomas, and AMLs. Increased c-Myc expressions alone or with additional Bcl-2 or Bcl-6 alterations correlate with poor clinical outcome, or therapy resistance [6,324,415,416,417]. Blocking c-Myc expression have been proven to interferes with tumor cell survival, motility and metastasis in various tumor models [50,410,418], with blocking mitochondrial pathways being an alternative or additional treatment possibility for tumors harboring *MYC* mutations or alterations resulting in overexpression.

### 3.9. Plasma Cell Myeloma, a.k.a Multiple Myeloma (MM)

#### 3.9.1. MM and Genetic Changes

MM is a neoplastic disorder of plasma cells, which are terminally differentiated B-cells. Numerous genetic alterations, including mutations and epigenetic changes, have been described in the dysregulation of plasma cells that lead to dyscrasias. Some significant genetic changes include *MYC*, *CCND1*/*CCND2*/*CCND3* (Cyclin D), *FGFR3* (fibroblast growth factor receptor 3), *MMSET* (histone methyltransferase multiple myeloma SET domain), *KRAS*, *NRAS*, *BRAF*, *TP53*, and *MAF*/*MAFA*/*MAFB* (musculoaponeurotic fibrosarcoma) [6,329,419]. According to the 2017 WHO Classification of Tumors of Hematopoietic and Lymphoid Tissue, *MAF* translocations (t14;16 and t14;20) and deletion of 17p (*TP53*), although relatively less common, portrays a poor prognosis [6]. Nonetheless, despite the broad knowledge of genetic abnormalities in multiple myeloma, dysregulation of mitochondrial functions has been less explored.

#### 3.9.2. MM and Necroptosis

As previously mentioned, since the initial discovery of necroptosis in the early 2000s, it has been increasingly investigated as a potential source of treatment for many conditions, including variety of neoplastic processes [420,421]. Prior studies on induction of necroptosis and mitochondrial fission in different malignancies, such as in multiple myeloma, had shown this to be a potential therapeutic target as seen by in vitro studies showing improved survival in multiple myeloma cells when regulators of fission were blocked [420].

#### 3.9.3. MM and Cellular Metabolism

Changes in cellular metabolism in MM are numerous, including a radical increase in glycolysis and OXPHOS. Enhanced glycolysis leads to higher pyruvate levels within the cell, which can be turned into lactate or acetyl-CoA as a substrate for the TCA cycle to go through OXPHOS. Acetyl-CoA can also be used for fatty acid synthesis. These processes are essential for neoplastic plasma cells in MM to proliferate and produce immunoglobulins in a stringent environment such as the bone marrow. The boosting of metabolic substrates and their subsequent processes within MM cells occur through numerous genetic changes that connect to the previously discovered changes occurring in MM discussed previously.

#### 3.9.4. MM, Cellular Metabolism and Effect of Genetic Changes 

Genetic alterations in *MYC*, *MAF*, *MMSET*, *FGFR3*, and *CCND1* lead to distinctly altered cellular metabolism through influence on enzymes, metabolic substrates, and various cellular transporters. The changes induced by these alterations enable enhanced cell survival and proliferation in MM. One of the main effectors in altered cellular metabolism is the protein kinase B cascade (AKT). This pathway plays a central role in multiple myeloma through metabolic orchestration leading to upregulation of glycolysis, glutaminolysis, pentose phosphate shunting, lipid synthesis, and nucleotide production while also decreasing fatty acid oxidation [329]. Furthermore, *AKT* is an essential inhibitor of FOXO, which is necessary for p53-dependent cell death. AKT is activated by cyclin D1, MYC, and FGFR3, while AKT further upregulates MMSET, which plays a pivotal role in opening chromatin for enhanced activity by demethylation. MMSET is also crucial for glycolytic processing and subsequent shunting through the pentose phosphate pathway (PPP). Interestingly, MMSET appears to be of particular importance in multiple myeloma cells containing the t(4;14) alteration. Cyclin D1 and MYC are stabilized by the activity of KRAS and ERK, while the activity of ubiquitin-specific peptidase 5 (USP5) helps prevent MAF degradation in t(4;14), t(11;14), t(14;16), and t(14;20) cells. Cancer lines with high levels of MAF are known to be resistant to proteasome inhibitors, such as bortezomib, through increased proteasomal activity. In addition, MYC appears to enhance both glycolysis and glutaminolysis in MM by acting on glutamine, lactate, and glucose transporters for augmented uptake from the environment [329].

#### 3.9.5. MM and Mitochondrial Dynamics and Mitochondrial Biogenesis

Interestingly, MM cell lines have demonstrated increased fusion, which is thought to be regulated by Myc [329]. In addition, high expression of the mitochondrial fusion gene *MFN1* is associated with inferior survival in MM [422]. On the other hand, mitochondrial fission, controlled by fission proteins Fis1 and Drp1, are necessary to handle a sudden rise in oxidative stress. To this same effect, multiple myeloma cells have been shown to induce the trafficking of mitochondria from neighboring stromal cells, which has been implicated in drug resistance development [1]. Furthermore, increased mitophagy has been described in MM, which can be related to drug resistance [1]. Increased expression of mtDNA translation, polymerase and helicase genes *TFAM*, *PGC1A, POLG2*, and *TWNK* have also been linked to poor prognosis in MM [422].

#### 3.9.6. Mitochondrial Transfer to MM Cells

Interestingly, MM cells are able to internalize mitochondria, transferred from the neighboring nonmalignant bone marrow stromal cells, which seems to be at least in part CD38-driven [423].

#### 3.9.7. MAF BZIP Transcription Factor (MAF)

Musculoaponeurotic fibrosarcoma (MAF) proteins, encoded by *MAF*, are basic region leucine zipper (bZIP)-type transcription factors. Large MAFs (lMAFs), such as *MAFA*, *MAFB*, *c-MAF* and *NRL* are pro-oncogenes, and bind to DNA sequences called *MAF* recognition elements (MAREs), where they modulate expression of several genes, such as *CCDN2*, *FOS*, *JUN*, *CREB, MTORC2* and *ATF* [329,424,425]. On the other hand, homodimers of small MAFs (*sMAF*), such as *MAFF*, *MAFG* and *MAFK* bind to the same region via competitive binding, inhibiting transcription. The ratio of lMAFs and sMAFs are therefore important determinants of transcription activation. In addition, posttranslational modifications also alter the activity of the MAF proteins [329].

MAF has multiple roles in cellular metabolism in MM, including elevated insulin secretion for enhanced MM cell glucose uptake, activating ARK5, which has been linked to increased OXPHOS, and glutamine metabolism. Glycolysis and glutaminolysis are of particular importance in MM due to the need for antibody production. The ARK5/CDK4 inhibitor ON123300 has been shown to induce apoptosis in MM cell lines. MAF translocations, however, were not predictive of ARK5/CDK4 treatment in this study [329,426].

In about 60% of angioimmunoblastic T-cell lymphomas, *c-MAF* was found to be overexpressed, whereas in MM, aberrant *c-MAF*, *MAFA,* and *MAFB* gene expressions have been described. As mentioned previously, *MAF* mutation is associated with a poor prognosis in MM [329,425,427].

## 4. Therapies Targeting Mitochondria

Here we aim to summarize potential therapies targeting mitochondria, some of which we already have addressed in previous chapters. In addition, specific therapies for malignancies with certain genetic mutations can be found in the chapters where those genes are discussed in detail.

### 4.1. Drugs Affecting Cellular Metabolism

Cellular metabolism can be inhibited via various enzymes in different metabolic pathways, which are promising therapeutic targets in cancer therapy. These include drugs targeting ETC, TCA, glycolysis, and fatty acid oxidation and synthesis enzymes. Some of these enzymes are localized in the mitochondria, whereas others are found in the cytosol (Figure 1 and Table 3) [7,428,429,430,431,432,433,434,435,436,437,438]. In addition, increasing mitochondrial ROS production is also an important mitochondria-related cancer therapy strategy [14,20]. For further details on these topics in relation to mitochondria see Section 2.1.

### 4.2. Drugs Affecting mtDNA Pathways and Cell Death Regulation

Inhibition of mtDNA translation and transcription [7,29,30,31], along with apoptosis [41,43,44,45,439] and necroptosis [440,441] induction are promising anti-cancer therapies (Figure 2 and Table 3), discussed in more details in Section 2.2 and Section 2.3.

### 4.3. Drugs Affecting Mitochondrial Dynamics

Interfering with mitochondrial dynamics, including mitochondrial fission/fusion [442,443], mitophagy [444], and mitochondrial trafficking opens up a large number of new therapeutic targets in cancer therapy (Table 3), discussed in more details in Section 2.4 and Section 2.5.

## 5. Conclusions

There is substantial evidence that genetic alterations in hematologic malignancies are in close relationship with mitochondrial metabolism, dynamics, cell death pathways, and mtDNA transcription and translation. Targeting these pathways are promising future therapies, especially in therapy-resistant cases.

## Figures and Tables

**Table 1:** Key oncogenic events in the pathogenesis of AML.

Class	Key Oncogenic Events
I. Proliferative and survival advantages	*FLT3, KIT, RAS, PNP11, JAK2, CBL, ERG, BAALC*
II. Alterations of cellular differentiation, apoptosis	*PML-RARA, RUNX1, RUNX1T1, CBFB, MYH11, MLL, CEBPA, NPM1, TP53*
III. No classification (recently described, mainly epigenetic modulators)	*DNMT3a, TET2, IDH1/2, ASXL1, WT1*

**Table 2:** The effects of transcription factors on mitochondria. Transcription factors described in detail in this review are summarized here with their main effects on mitochondrial metabolism, dynamics, and apoptosis. In addition, the involvement in various hematologic malignancies is also summarized. ↑: increase, ↓: decrease. Abbreviations: AML: Acute myeloid leukemia, BL: Burkitt lymphoma, DLBCL: Diffuse large B-cell lymphoma, Drp1: dynamin-related protein1, GLS2: mitochondrial glutaminase 2, MCL: Mantle-cell lymphoma, Mff: mitochondrial fission factor, lMAFs: large Musculoaponeurotic fibrosarcoma (MAF) proteins, MDS: Myelodysplastic syndrome, MM: Multiple myeloma, OXPHOS: oxidative phosphorylation, ROS: reactive oxygen species, sMAF: small Musculoaponeurotic fibrosarcoma (MAF) proteins.

Gene	Coded Protein	Effect on Nuclear DNA	Metabolic Role	Mitochondrial Role	Oncogenic Effect
*MYC* family: *MYC* *MYCN* *MYCL*	c-Myc	p53 activation	Expression of GLUT1, 3↑ Hexokinase activity ↑ Expression of lactate, glutamine transporters↑ Nucleotid synthesis ↑ Amino acid synthesis ↑ Fatty acid synthesis ↑ β-oxidation ↑ Glycolysis ↑ Glutaminolysis ↑	ROS generation ↑ OXPHOS ↑/↓ Expression of mitochondrial trafficking proteins (RHOT1, RHOT2, TRAK2, and Kif5B) ↑ Fission (Drp1, Mff) ↑ Mitochondrial recruitment to cortical cytoskeleton ↑ Organelle movements ↑ Mitochondrial biogenesis and replication (*PLOG*, *PLOG2*, and *NRF1* expression) ↑	BL, Double hit/triple hit DLBCL, MM, MCL, AML
*RUNX1*	RUNX1/ RUNX1T1 fusion protein	Local remodeling of chromatin	Cell differentiation ↓ Self-renewal ↑ (unknown mechanism)	Apoptosis ↓ Fission (Drp1-dependent) ↑ ROS production ↑	Granulopoiesis, Dysplasia- MDS phenotype, Development of post MDS-AML
*TP53*	Tumor protein 53	MYC-TP53 cross talk Topoisomerase IIα stabilization, Transcriptional inhibition of PFKFB3 and PFKFB4 genes, PDK2 transcription ↓	Autophagy ↑ PUMA, NOXA ↑ GLUT 1,3,4 ↓ Hexokinase 2, Phosphoglycerate mutase 1 ↓ Parkin ↑ HIF1α degradation Fructose-2,6-bisphosphate expression ↓ =promoting glycolysis Pentose-phosphate-pathway ↓ Fatty acid synthesis ↓ Lipid synthesis ↑	Apoptosis ↑: Bcl2/BclIX ↓ Bax/Bak ↑ Cytochrome C release ↑ Opa1 cleaving ↑ OXPHOS ↑: Mitochondrial GLS2 ↑ Drp1 blocking-highly interconected mitichondria ↑ PKA activation-mitochondrial elongation Fission ↑ ROS synthesis ↓	Li-Fraumeni syndrome, MDS, AML
*RAS* family: *HRAS* *NRAS* *KRAS*	small GTPases	Replication stress RAS/MYC functional connection Accelerated transcription via TBP	Major downstream RAS effector pathways: phosphatidylinositiol-3-kinase (PI3K), mitogen-activated protein kinase (MAPK), Ras-like (Ral) small GPTase signaling pathways Glycolysis ↑ (via STAT3)	OXPHOS ↓(via STAT3) ROS synthesis ↑ Fission (Drp1-dependent) ↑	AML, MDS
*MAF*	MAF proteins lMAFs (oncogenes): MAFA, MAFB, c-MAF, NRL sMAFs *(transcriptional factors):* MAFF, MAFG, MAFK	*lMAFs:* CCDN2, FOS, JUN, CREB, MTORC2, ATF expression modulation *sMAFs*: inhibiting transcription	insulin secretion ↑ cell glucose uptake ↑ glutamine metabolism ↑	OXPHOS ↑ Apoptosis ↑	MM, Angioimmunoblastic T-cell lymphoma

**Table 3:** Summary of drugs targeting mitochondria and glycolysis.

Group of Drug	Drug/s	Mechanism of Action
Mitochondrial metabolism:ETC inhibitors	Metformin IACS10759 BAY87-2243 MitoTam	Respiratory Complex I inhibition [7,428]
	MitoVES	Respiratory Complex I-II inhibition [428]
	Lonidamine	Respiratory Complex II inhibition [428]
	ME344	Respiratoy Complex I-IV inhibition [7,432]
	VLX600	ETC inhibitor [428]
Mitochonrial metabolism:TCA enzyme inhibitors	CPI-613 (devimistat)	PDH and KGDH inhibition [7,428]
	Enasibenib Ivosibenib	IDH inhibition (mutated IDH) [7,428]
Metabolism: glycolysis and other pathway inhibitors	Dichloroacetate (DCA)	PDH kinase inhibitor [428,430]
	WZB117 STF31 Phloretin Quercetin	GLUT1 inhibitors [431]
	2-DG (2-Deoxy-D-glucose) 2-FDG (2- fluorodeoxy-D- glucose)	Competitors for binding hexokinase (converting glucose to glucose-6-phosphate) [428]
	3-Bromopyruvate	Hexokinase inhibitor [431]
	Diclofenac Lumiracoxib	Anti-inflammatory drugs with glycolysis inhibition [428]
	3PO PFK-158	Phosphofructokinase inhibition (phosphofructokinase converts fructose-6-P to fructose-1,6-bisP), inhibiting glycolysis [431,434,435]
	Oxamic acid NHI1 1-(Phenylseleno)-4-(trifluoromethyl)benzene Gossypol	Lactate dehydrogenase inhibitor (lactate dehydrogenase converts pyruvate to lactate), inhibiting glycolysis [428,431,433]
	CB-839 Compound 27 968 BPTES (bis-2-(5-phenylacetamido-1,2,4-thiadiazol-2-yl)ethyl sulfide)	Glutaminase inhibitor (converts glutamine to glutamate) [7,14]
	L-Asparginase	Glutamine depletion [14]
	Sulfasalazine Erastin	Glutamine-cystine antiporter (xCT, a heterodimer of SLC7A11 and SCL3A2) inhibitor [14]
	Gamitrinib	Heat shock protein 90 (HSP90) inhibitor, inhibiting various metabolic pathways [428,429]
	Etomixir	carnitine palmitoyltransferase-1 (CPT-1) inhibition (fatty acid oxydation inhibitor) [437]
	C75 Cerulein Orlistat Triclosan Amentoflavone EGCG TVB-3166	Fatty acid synthase inhibitors [438]
Increased ROS production	Cisplatin 5-FU Paclitaxel Procarbazine	ROS production [7,20]
mtDNA transcription and translation inhibition	IMT1 IMT1B	Mitochondrial RNA polymerase (POLMRT) inhibition (transcription inhibition), interfering with ETC protein transcription [29]
	Tigecycline	mtDNA translation inhibition [7,30,31]
Apoptosis inductors	Venetoclax (ABT-199) BCL201 (aka S55746)	Bcl-2 inhibitors [45,439]
	BTSA1	Bax activator [43]
Necroptosis inhibition	TAK-632 Ponatinib	RIP1 and RIP3 inhibitiors [440,445] (with TAK-632 also being a pan-RAF inhibitor [441])
	Nec-1 GSK2982772 (Compound 5) RIPA-56 (Compound 56) 7-oxo-2,4,5,7-tetrahydro-6*H*-pyrazolo [3,4-c]pyridine (Compound 22) Tozasertib (a.k.a. VX-680 and MK-0457) Pazopanib	RIP1 inhibitors [445]
	Dabrafenib	RIP3 and B-Raf inhibitor [445]
Drugs altering mitochondrial dynamics	Mitochondrial division inhibitor-1 (Mdivi-1) Drpitor1 and Drpitor1a	Mitochondrial fission inhibitors [442,443]
	Melatonin Mdivi-1 Liensinine	Mitophagy inhibitors (helping downregulating drug resistance in certain tumors) [444]
	Ketaconazole Sorafenib Mito-CP and Mito-Metformin	Mitophagy inducers (leading to apoptosis due to insufficient number of mitochondria) [444]

*Abbreviations*: ETC: electron transport chain, IDH: isocitrate dehydrogenase, KGDH: α-ketoglutarate dehydrogenase, mtDNA: mitochondrial DNA, PDH: pyruvate dehydrogenase, ROS: reactive oxygen species, TCA: tricarboxylic acid cycle.

## Supplementary Materials

The following are available online at https://www.mdpi.com/article/10.3390/life11121351/s1, Table S1: Abbreviations.
