# Supplementary material for: Mitochondria and Their Relationship with Common Genetic Abnormalities in Hematologic Malignancies
Source: Life (Basel). 2021 Dec 7;11(12):1351. doi: 10.3390/life11121351 (PMC8707674; doi:10.3390/life11121351)
Supplement: Supplementary file 1 [file life-11-01351-s001.zip › life-1467459-supplementary.pdf]

life

Supplementary Material Mitochondria and Their Relationship with Common Genetic Abnormalities in Hematologic Malignancies

Table S1. Abbreviations.

2-DHG	2-dihydroxyglutarate
5mC	5-mehtylcytosine
α -KG	α-ketoglutarate
ABCB7	ATP Binding Cassette Subfamily B Member 7
ABC DLBCL	DLBCL, activated B-cell type
ABVD	doxorubicin, bleomycin, vinblastine, and dacarbazine therapy
ACACA	acetyl-coenzyme A carboxylase alpha
ALL	acute lymphoblastic leukemia
Ambra1	activating molecule in BECLIN1-regulated autophagy
AML	acute myeloid leukemia
AMPK	adenosine monophosphate -activated protein kinase
ASCT	autologous stem cell transplantation
ASXL1	ASXL Transcriptional Regulator 1
ATO	arsenic trioxide
ATP	adenosine triphosphate
ATRA	all-trans retinoic acid
AZA	azacytidine CEBPβ
BAP1	BRCA1-associated protein 1
B-ALL	B acute lymphoblastic leukemia
Bcl-2	B-cell lymphoma 2
BCR	B-cell receptor
Bnip3	Bcl-2 adenovirus E1B 19 kDa-interacting protein 3
CBFB	Core-Binding Factor, Beta Subunit
CBL	Casitas B-lineage Lymphoma
CCAAT	enhancer-binding protein β
CEBPA	CCAAT Enhancer Binding Protein Alpha)
cHL	classical Hodgkin lymphoma
CLL	chronic lymphocytic leukemia
CML	chronic myeloid leukemia
CMML	chronic myelomonocytic leukemia
CpG	regions of DNA where a cytosine nucleotide is followed by a guanine nucleotide
DLBCL	Diffuse large B-cell lymphoma
DLST	dihydrolipoamide S-succinyltransferase
DNMT	DNA-methyltransferase family
Drp1	dynamin-related protein1
DNAm	DNA methylation
ER	endoplasmic reticulum
ERK	extracellular regulated protein kinases 1/2
ETC	electron transport chain
EZH2	enhancer of zeste homolog 2
FGFR3	fibroblast growth factor receptor 3
Fis1	fission protein 1

FL	follicular lymphoma
FLT3	fms-like tyrosine kinase 3
FOXO3a	Forkhead box O3a
Fundc1	FUN14 Domain Containing 1
GATA2	GATA-binding factor 2
GCB DLBCL	DLBCL, germinal center B-cell type
GLS2	mitochondrial glutaminase 2
HDAC	histone deacetylase
HIF- Prolyl hydroxylase	hypoxia inducibe factor prolyl hydroxylase
HL	Hodgkin lymphomas
HMA	hypomethylating agent
HSC	hematopoietic stem cell
IDH	isocitrate dehydrogenase
IMM	inner mitochondrial membrane
JAK2	Janus Kinase 2 gene
JmjC-HD	JmjC domain-containing histone demethylase
JNK	c-Jun N-terminal kinase
KGDH:	α-ketoglutarate dehydrogenase
KIT	receptor tyrosine kinase type III
KRAS	gene Kirsten rat sarcoma viral oncogene homolog
LDH	lactate dehydrogenase
lMAFs	large MAFs
MAF	musculoaponeurotic fibrosarcoma
MCL	mantle cell lymphoma
MAM	mitochondria associated endoplasmic reticulum membrane
MAPK/ERK1/2	mitogen-activated protein kinase/extracellular signal-regulated protein kinases 1/2
MDS	myelodysplastic syndrome
MDV	mitochondria derived vesicle precursors
Mff	mitochondrial fission factor
Mfn1/2	mitofusin 1/2
MIEFs	mitochondrial elongation factors
MLL	mixed lineage leukemia
MLKL	mixed lineage kinase domain-like pseudokinase
MM	multiple myeloma
MMSET	histone methyltransferase multiple myeloma SET domain
MOM	mitochondrial outer membrane
MOMP	mitochondrial outer membrane potential
MPN	Myeloproliferative neoplasms
MRC-AML	AML with myelodysplasia related changes
mtDNA	mitochondrial DNA
mTORC1	mammalian target of rapamycin complex 1
MYH11	Smooth muscle myosin heavy chain locus 11
MYO	Myosin motor
mTERF1	mitochondrial termination factor
NADP+/NADPH	Nicotinamide adenine dinucleotide phosphate
nDNA	nuclear DNA
nHL	Non-Hodgkin lymphoma
Nix	Nip3-like protein X or Bnip3L
NK cell	natural killer cell

NLP-HL	nodular lymphocyte-predominant Hodgkin lymphoma
NLRP3	NOD-, LRR- and pyrin domain-containing protein 3
NPM1	nucleophosmin gene
NRAS	Neuroblastoma RAS viral [v-ras] oncogene homolog
OMM	outer mitochondrial membrane
OPA1	Optic atrophy 1 protein
OXPHOS	oxidative phosphorylation
PGC-1α	peroxisome proliferator-activated receptor gamma coactivator 1-alpha
PI3K/Akt	phosphoinositide-3-kinase/ protein kinase B
PINK1	phosphatase and tensin homologue (PTEN)-induced putative kinase 1
PKA	protein kinase A
PML-RARA	Promyelocytic leukemia/retinoic acid receptor
PNP11	protein tyrosine phosphatase, non-receptor type 11
POLG	mtDNA polymerse gamma
POLRMT	mitochondrial RNA polymerase
PPM1D	Protein phosphatase Mg2+/Mn2+ dependent 1D
PPP	pentose phosphate pathway
PRC1	polycomb repressive complex 1
PTCL-NOS	peripheral T-cell lymphoma, not otherwise specified
R-2-HG	R-2-hydroxyglutarate
RAEB	Refractory Anaemia with Excess Blasts
RARS	refractory anaemiawith ring sideroblasts
R-CHOP	rituxiban-cyclophosphamide, hydroxydaunorubicin, Oncovin, prednisone treatment
RGL1	ral guanine nucleotide stimulator like 1
RHD	RUNT homology domain
RIP1/3	receptor-interacting proteins 1
ROS	reactive oxygen species
r/r/ HL	relapsing/refractory Hodgkin lymphoma
rRNA	ribosomal RNA
RS cells	Reed-Sternberg cells
RUNX1	Runt-related transcription factor 1
SAM	S-adenozyl-methionine
SF3B1	Splicing factor 3b subunit 1
shRNA	short hairpin RNA
sMAFs	small MAFs
SNPH	syntaphilin
SRSF2	Serine and arginine Rich Splicing Factor 2
STAG2	stromal antigen 2
STAT3	signal transducers and activators of transcription 3
t-AML	therapy-related AML
TCA	tricarboxylic acid cycle
TET	metylcytosine dioxygenases of the Ten Eleven Translocation superfamily
TP53	tumor protein 53
tRNA	transfer RNA
trxG	trithorax group activator complex
TEFM	transcription elongation factor
TFAM	mitochondrial transcription factor A
TFB1M/2M	mitochondrial transcription factor B1/2
U2AF1	U2 small nuclear RNA auxiliary factor 1

VEN	venetoclax
TP53	tumor protein 53
TWNK	twinkle mtDNA helicase
WT	wild type
WT1	Wilms' tumor 1 gene
ZRSR2	U2 small nuclear ribonucleoprotein auxiliary factor 35 kDa subunit-related protein 2
